# Composition-dependent nanoelectronics of amido-phenazines: non-volatile RRAM and WORM memory devices

**DOI:** 10.1038/s41598-017-13754-w

**Published:** 2017-10-17

**Authors:** Dilip K. Maiti, Sudipto Debnath, Sk. Masum Nawaz, Bapi Dey, Enakhi Dinda, Dipanwita Roy, Sudipta Ray, Abhijit Mallik, Syed A. Hussain

**Affiliations:** 1 Department of Chemistry, University of Calcutta, 92 A. P. C. Road, Kolkata, 700009 India; 2 Department of Electronic Science, 92 A. P. C. Road, Kolkata, 700009 India; 3 Department of Physics, Tripura University, Suryamaninagar, 799022, Tripura, India

## Abstract

A metal-free three component cyclization reaction with amidation is devised for direct synthesis of DFT-designed amido-phenazine derivative bearing noncovalent gluing interactions to fabricate organic nanomaterials. Composition-dependent organic nanoelectronics for nonvolatile memory devices are discovered using mixed phenazine-stearic acid (**SA**) nanomaterials. We discovered simultaneous two different types of nonmagnetic and non-moisture sensitive switching resistance properties of fabricated devices utilizing mixed organic nanomaterials: (a) ***sample-1***(**8**:**SA** = 1:3) is initially off, turning on at a threshold, but it does not turn off again with the application of any voltage, and (b) ***sample-2*** (**8**:**SA** = 3:1) is initially off, turning on at a sharp threshold and off again by reversing the polarity. No negative differential resistance is observed in either type. These samples have different device implementations: ***sample-1*** is attractive for write-once-read-many-times memory devices, such as novel non-editable database, archival memory, electronic voting, radio frequency identification, ***sample-2*** is useful for resistive-switching random access memory application.

## Introduction

The leading solid state memory technologies, dynamic random access memory (DRAM) and flash memory, suffer from major short comings that may challenge the rapid growth of high-speed, low-cost, and low-power computational systems. Although DRAM is capable of operating at moderate speed, it consumes additional power, as it requires cyclic refreshment of stored information. Despite its non-volatile data retention characteristics, flash memory technology is not suitable for ultra-high speed applications because of its significantly long write/erase time and limited endurance. These limitations of solid state memory technologies have triggered both academic and industrial quests for the development of novel memory technologies, which should possess the speed of DRAM and the non-volatility of flash memory. The innovative memory technologies explored to date have been mainly focused on some metal-based materials that are capable of switching their intrinsic properties depending on their operating conditions^1–4^. Among the emerging memory technologies, resistive-switching random access memory (RRAM)^5–18^, which utilizes the switching of resistance states for data storage, shows great promise. For another important non-volatile memory device, which is also based on the resistive-switching (RS) phenomenon, write-once-read-many-times (WORM) operation is desirable for permanent data storage applications, such as a non-editable database, archival storage of images, electronic voting, and radio frequency identification (RFID),where magnetic or optical disk drives are not acceptable due to their large size, vulnerability to breakage, relatively high cost, low speed of operation, and large power consumption. The enormous utility of resistive-switching memory performances has led to the development of a few new materials in recent times, including amorphous Si^5,6^, inorganic metal oxides^7–12^, metal/NP-polymers^13–18^, virus-NPs^19^, silver-electrolytes^20^, organometallic complexes^21^, nylon-graphene-polymers^22^,multi-walled carbon nanotubes^23^, and fullerene-polyethylene^24^. Organic electronics^25–30^, based on innovative organic nanomaterials consisting of small molecules that possess noncovalent gluing attractive forces, feature easy formation of light weight large-scale fabrication, more flexibility, biodegradability and less expensive materials through unidirectional packing of nanobuilding blocks.These nanomaterials^28,29^ are potential candidates for use in devices with much improved semiconducting, memory, and storage device performances. More importantly, organic nanomaterials display no magnetic interference, and their electronic properties can easily be tuned via simple structural modification of the nanobuilding blocks, which are practically impossible to achieve in inorganic electronics. Thus, designing new organic nanobuilding blocks through installation of attractive noncovalent attractive forces, developing a simple strategy for their synthesis, fabricating unidirectional packing nanomaterials and discovering new electronic property are desirable tasks for achieving organic electronic-based high-tech devices of ultimate sensitivity. Recently Zhang^30^ and Xu^31^ coworkers reported preliminary RRAM and WORM properties using imidazole and hydroxyl substituted phenazine derivatives, respectively. In this manuscript, we have demonstrated for the first time that by varying the mixture ratio of newly designed and synthesized amido-phenazine and SA, both RRAM and WORM are achievable.

**Figure 1:**
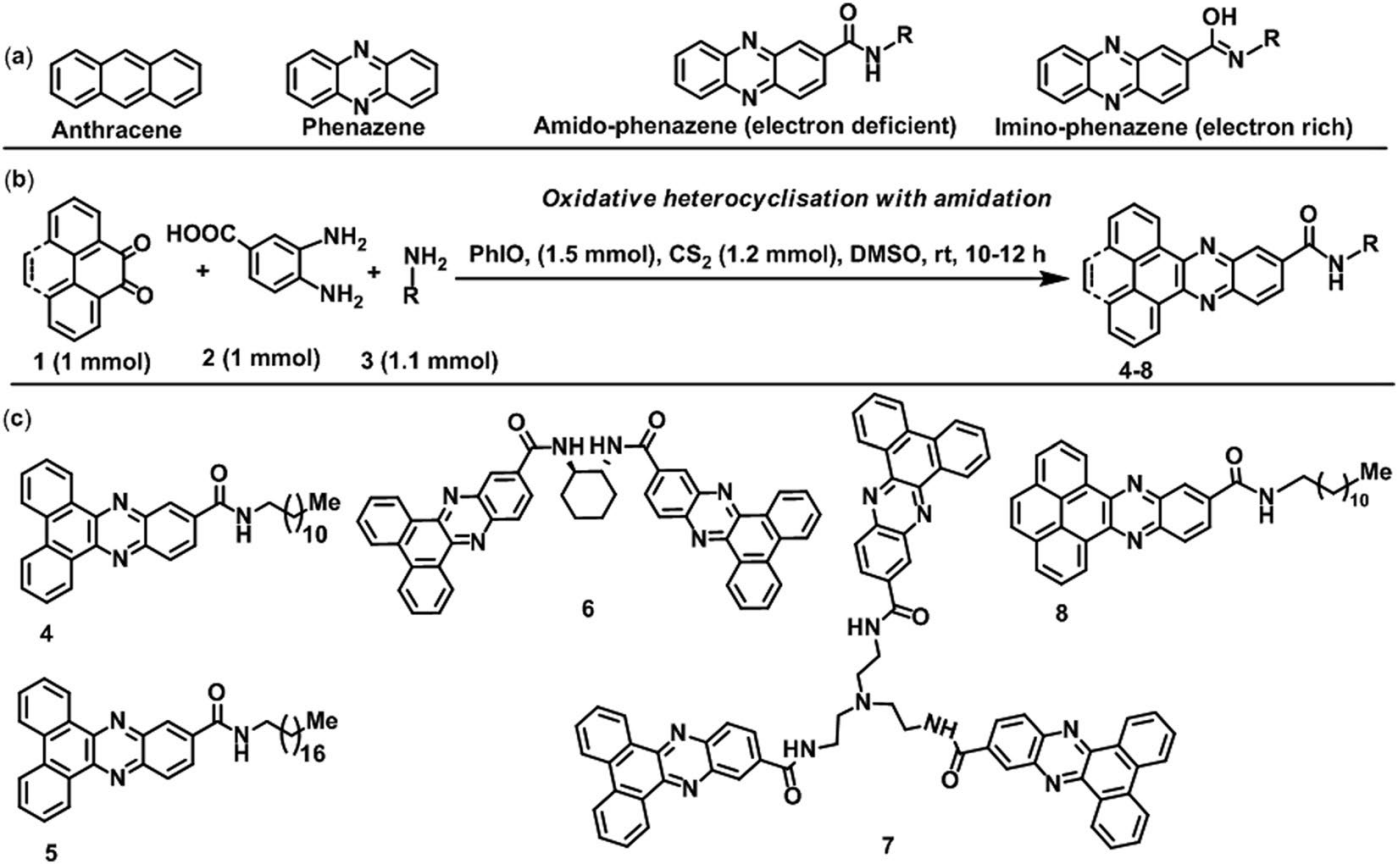
(**a**) Anthracene, phenazine aromatic frameworks, pure and enolized forms of amido-phenazines. (**b**) General direct syntheses of phenazine nanobuilding blocks. (**c**) Synthesized phenazine compounds.

We envisaged unsymmetrical electron-rich phenazine compounds bearing chemically stable and electron deficient amide functionality as the ideal nanobuilding blocks for the development of the next-generation memory materials. The amide functionality in the phenazines can be utilized to produce a more electron deficient nanobuilding framework as electrically resistive materials, which can easily be modified into conducting nanomaterials through change of polarization involving conversion of the amide group into an electron donating group upon application of a higher voltage or use of a suitable external molecule (Fig. 1a). An optically inert long chain fatty acid **SA** ^32^ may be used as a stabilizing matrix compound to achieve a mixed component system for the development of a wider variety of properties compared to the material of pure phenazine and to determine the thermodynamic behavior of the constituent molecules for specific electronic applications. Alternatively, the replacement of two =CH- by the =N- moiety of the frequently used polynuclear π-conjugated aromatic hydrocarbon anthracene (Fig. 1a) drastically changes the nuclear electron deficiency, aggregation ability, energies of the HOMO and LUMO, band gap and desirable charge transport behavior of our designed phenazine compounds.

Natural phenazine compounds and their synthetic analogues have been detected in the following forms: anti-respiratory secretion^33^,antibiotic, antitumor, antimalarial and antiparasitic compounds^34^, microorganisms^35^, anticancer prodrug^36^, insecticide^37^, and ruthenium-based DNA-intercalation and aggregate-detector^38^, colorimetric^39^, photophysical^40^, and showed preliminary observation for material application^41–43^. Only a limited number of synthetic methods are found in the literature, such as synthesis through functionalization of the phenazines frameworks^41^, RuCl_3_-catalysed cyclization of aromatic azides^44^, coupling of 1,2-diketones with 1,2-diamines^37,45^ and oxidative coupling of amines^38^.

### Direct synthesis of phenazines and thermodynamic behavior in thin films

Bearing in mind the pharmaceutical and possible organic electronic applications of phenazines, a metal-free unprecedented three component cyclization of aromatic 9,10-phenanthroquinone (**1a**), 3,4-diaminobenzoic acid (**2a**) and dodecylamine (**3a**) based on the amidation strategy is established using Lewis acid-like oxidant PhIO and carbon disulfide for easy synthesis of designed phenazine dodecyl amide (**4**, Fig. 1b) at ambient temperature. Herein, the striking difference from previous cyclization strategies is that the essential extra aromatic ring, chemically stable functionalities, a tunable hydrocarbon chain, a variable chain length, and thermally labile chirality can easily be installed simultaneously under mild conditions (Fig. 1c). The substrate scope of the new reaction was validated through synthesis of another monopodal phenazine bearing octadecyl amide (**5**), chiral dipodal phenazine (**6**), tripodal phenazine (**7**) and *N*-dodecylphenanthro[4,5-*abc*]phenazine-11-carboxamide (**8**). All synthesized new molecules were fully characterized by related spectroscopic measurements and other methods (Supplementary Information).

**Figure 2:**
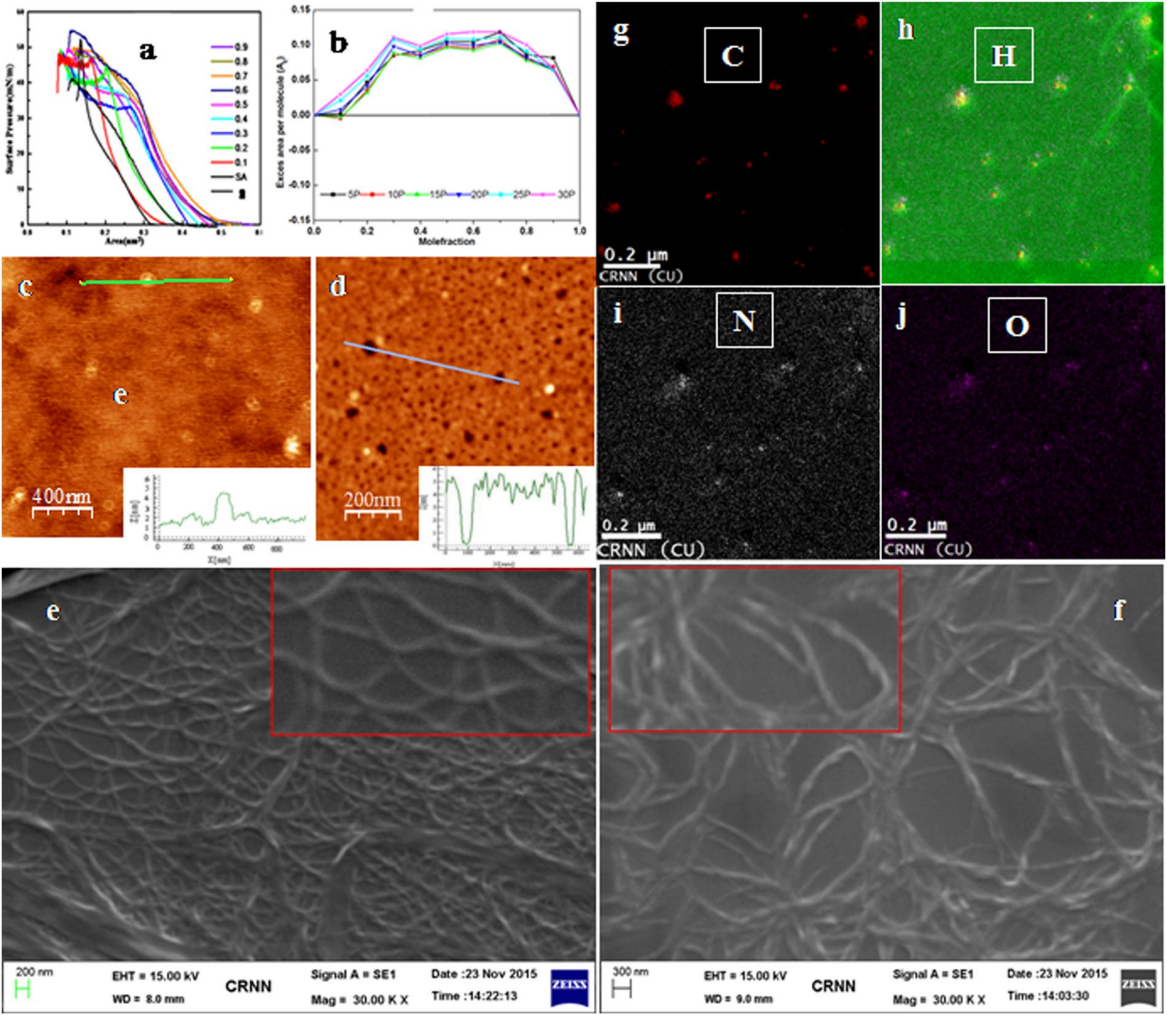
(**a**) Surface pressure (π) versus area per molecule (A) isotherms of **8** and **SA** at different mole fractions of **8** along with pure **8** and **SA** isotherm (numbers denoting corresponding mole fractions of **8** and **SA** matrix at air-water interface). (**b**) Plot of excess area per molecule (A_E_) vs the mole fraction of **8** in the dilute-SA mixed monolayer at surface pressures of 5, 10, 15, 20, 25, and 30 mN/m. (**c**) AFM image of monolayer LB film of pure phenazine **8**. (**d**) Mixed with **8**-**SA** at mole fraction X_8_ = 0.3 on to silicon substrate. (**e**) SEM image of ***sample 1*** (**8**:**SA** = 1:3). (**f**) SEM image of ***sample 2*** (**8**:**SA** = 3:1). (**g**–**j**) TEM-EELS mapping images.

We determined the monolayer stability characteristics of pure phenazine (**8**), **SA** and mixed **8**-**SA** Langmuir films at the air–water interface using different mole fractions (Supplementary Information). The pure **SA**-isotherm (*π*–*A*) was consistent with that reported^46^. The initial higher lift-off area for the pure **8** bearing bulky head group (Fig. 2a) was due to the larger head group of **8** compared to **SA**, and the mixed monolayer isotherms of **8-SA** were beyond the monolayer molecular areas of the pure **8** and **SA** isotherms showing positive excess area (A_E_), which confirmed the strong binding ability between **8** and **SA** (X_**8**_ = 0.1–0.3). Interestingly, the binding interaction became optimum at the mole fraction 0.3 (i.e., **8**:**SA** = 1:3) and was independent of mole fraction with higher mole fractions (>0.3 to <0.9, Fig. 2b) due to the constant positive deviation. The results obtained from the surface phase rule by calculating the Gibb’s excess free energy of mixing (Δ*G*_*exc*_) were in good agreement with that of A_E_ (Supplementary Information). We investigated Brewster Angle Microscopy (BAM) experiments (Supplementary Information) using pure **8** and **SA-8** mixed monolayer. The BAM images of mixed monolayer showed that domains are formed due to interaction between the constituent **8** and **SA** molecule in the mixed films. However nature of domain formation is little bit different for lower and higher mole fraction of **8**. This is because at lower mole fraction (0.1–0.3) strong binding ability between **SA** and **8** molecules is observed (Fig. 2b), whereas, mole fraction greater than 3 interaction between **SA** and **8** molecules balance each other. As a whole BAM study give compelling visual evidence of the formation of monolayer at air-water interface.

### Design, fabrication, imaging and UV-Vis study

Our density functional theory (DFT) study utilizing Gaussian 09 software^47^ enabled us to find out compound **8** possessing band gap 0.1210 eV, polar nature (dipole moment 4.0823 Debye) and the expected molecular packing of higher order due to the presence of strong noncovalent gluing interactions such as hydrogen bonding and π−π stacking (Supplementary Information). The synthesized phenazines (**4**–**8**) possessing lower symmetry elements and several noncovalent weak binding forces are instrumental in the construction of unidirectional packing nanomaterials, such as rods (**4**), flowers (**5**), flake-assemblies (**6**), nanoballs (**7**, spin coating), ultra-long nanofibrils (**8**, drop-casting) and small fiber (mixed **8**:**SA** = 1:1, drop casting)-like one-dimensional (1D) morphologies found in scanning electron microscope (SEM) imaging (Supplementary Information).

AFM images presented herein are the topographic image of the pure **8** LB monolayer (Fig. 2c) and **8-SA** mixed LB monolayer (Fig. 2d). Colour in the topographic image represents the height, where white corresponds to maximum height and dark corresponds to minimum height. From Fig. 2c and the height profile analyses, it is seen that pure **8** formed smooth monolayer film with almost uniform thickness with surface roughness ranging between 0–0.5 nm and the overall thickness of the film is 2 nm. The height of white spot seen in the image is approximately 4.5 nm and diameter is of the order of 90 nm. This corresponds to the cross sections of the ultra-long nanofibrils formed in the film as seen in the SEM image (Supplementary Information). On the other hand, the AFM image and height profile of the mixed **8**-**SA** LB film shows formation of nanoporous structure with almost uniform surface coverage. The kinds of structures are formed due to the repulsive interaction between the **SA** and **8** molecules in the mixed monolayer as confirmed from the excess area analysis (Fig. 2b). The black spot on the image corresponds to the nano pore formed in the mixed films. From the height profile analysis it is seen that the maximum width of the pore ranges from 40–50 nm. To our delight, the AFM image of the mixed **8**-**SA** LB film exhibited fabrication of nanoporous materials of almost uniform surface coverage (Fig. 2d). As a result, **8**-**SA** LB is an ideal material for high performance organic nanoelectronic devices. The photophysical studies of the pure **8** and mixed **8**-**SA** LB films showed red-shifted and broadened UV-vis spectra, which confirmed the existence of J-type aggregation (Supplementary Information). The unique thermodynamic behavior of Langmuir films (Fig. 2b) inspired us to achieve fabricated nanomaterials of composition 1:3 (***sample 1***) for robust memory applications. Scanning electron microscope (SEM) imaging of the mixed material of ***sample 2*** (3:1) revealed formation of nanofibrils of smooth surface (Fig. 2e and inset), where as ***sample 1*** (1:3) had roughness in its nanofibrils structure (Fig. 2f and inset). To understand the composition of nanofibrils we captured transmission electron microscope (TEM) images of highly diluted spin coated ***sample 1*** (1:3), which revealed existence of spherical nanomaterials of ~10 nm dimension. ***Sample 2*** (3:1) showed construction of little smaller nanoparticles (~ 5 nm) with less crystalline in nature (Supplementary Information). These composition dependent structural changes at the nanoscale may lead to development of new organic nanoelectronics for the two types of materials. TEM-EELS^48^ mapping of the mixed material confirmed the presence of C, H, N and O as constituting atoms (Fig. 2g–j).

### Fabrication of non-volatile memory device

**Figure 3:**
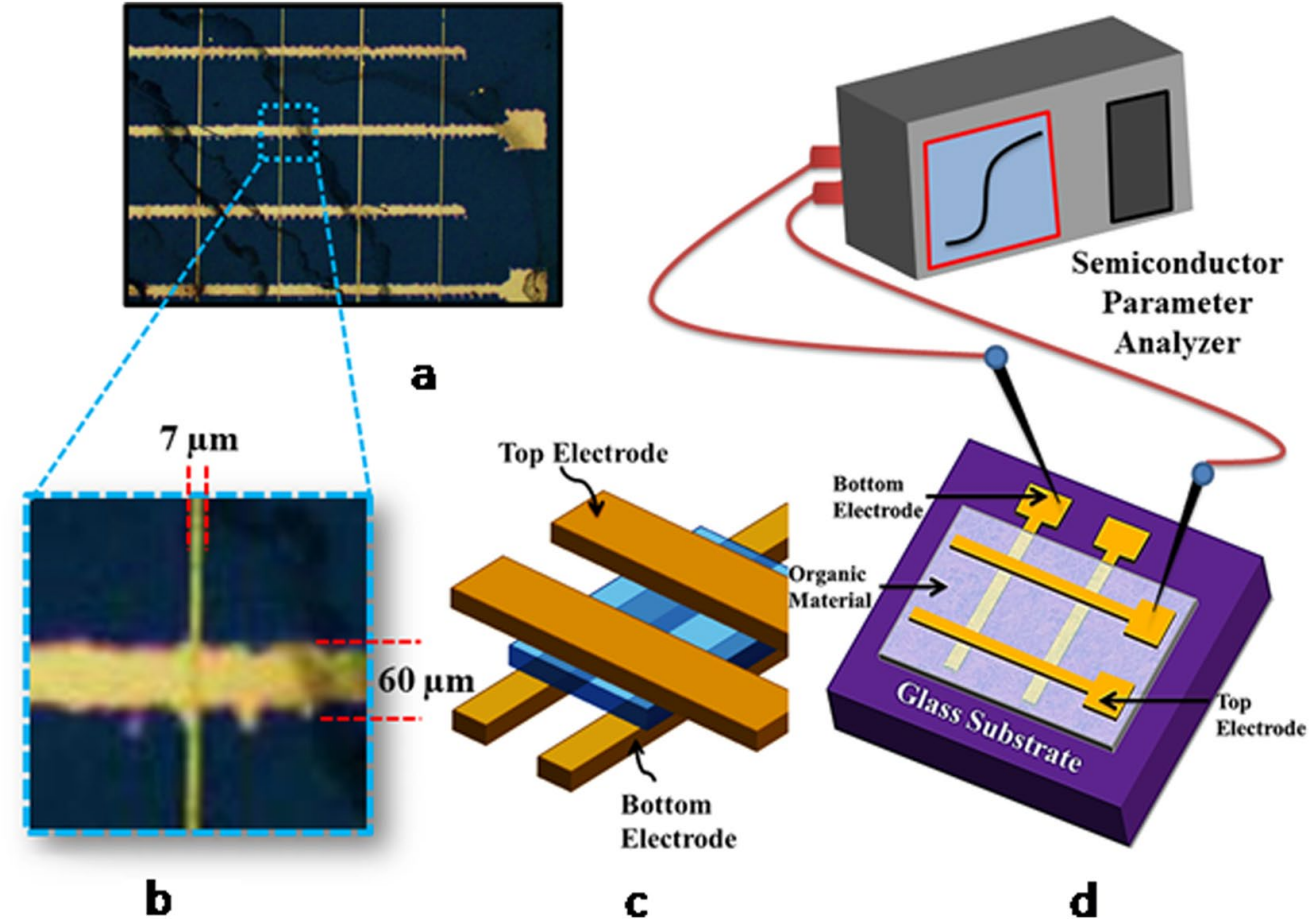
(**a**) Optical image of the fabricated cross-bar device. (**b**) Enlarged device with area 7 µm × 60 µm. (**c**) 3D Bird’s-Eye view of the schematic crossbar RRAM. (**d**) Measurement setup.

We fabricated the cross-bar device structure using cleaned glass substrates in piranha solution; the schematic device structure with the complete electrical characterization setup is depicted in Fig. 3a. Lithography followed by lift-off was performed to pattern the bottom electrodes (BE); the photoresist mold that defines the bottom electrode pattern was first created on the clean glass substrate. The resist nanomaterials were fabricated by spin coating with an initial revolution at 500 rpm for 10 s and then 2500 rpm for 40 s on the glass substrate. Next, the resist was baked on a hot plate at 85 °C for 60 s. A mask aligner system was used to expose the resist to UV light through a photomask for 9 s at 9.25 mW/cm^2^. Post-exposure baking at 85 °C for 60 s was also performed. The photoresist was developed, subsequently cleaned in isopropyl alcohol and then dried with nitrogen blow. A combination of 20 nm thick Cr/100 nm thick gold (Au) was thermally evaporated at a vacuum level of 5 × 10^−6^ Torr on the substrate containing the mold. Because Au has inadequate adhesion to glass, a thin layer of Cr was used in combination with Au. Patterning of BE was completed after removal of the remaining Cr/Au from the region outside the mold by using a lift-off process. In the next step, the organic material (**8**:**SA** = 1:3) was spin-coated on the surface of the substrate that contained the BE. Prior to spin coating, the substrates were again cleaned in acetone. Finally, Au top electrodes (TEs) were thermally evaporated at a vacuum level of 4 × 10^–6^ Torr on the organic layer through a shadow mask, which was aligned to the BE at an angle of 90° to complete the fabrication of the cross-bar device structure. Two different line widths of 5 µm and 10 µm were chosen for the bottom electrodes; the top electrodes were kept fixed at 60 µm (Fig. 3b). Because each cross point where the organic layer is sandwiched between bottom and top electrodes defines a device, two different device areas of 5 µm × 60 µm and 10 µm × 60 µm were formed for the Au/**SA**:**8**/Au device structure fabricated in this experiment. Electrical characterization of the devices was performed using a semiconductor parameter analyzer under ambient conditions. Voltage is applied on the TE (V_TE_) with a compliance current of 10 mA, while the BE is always kept grounded for all the devices.

### *Sample-1*: WORM characteristics

**Figure 4:**
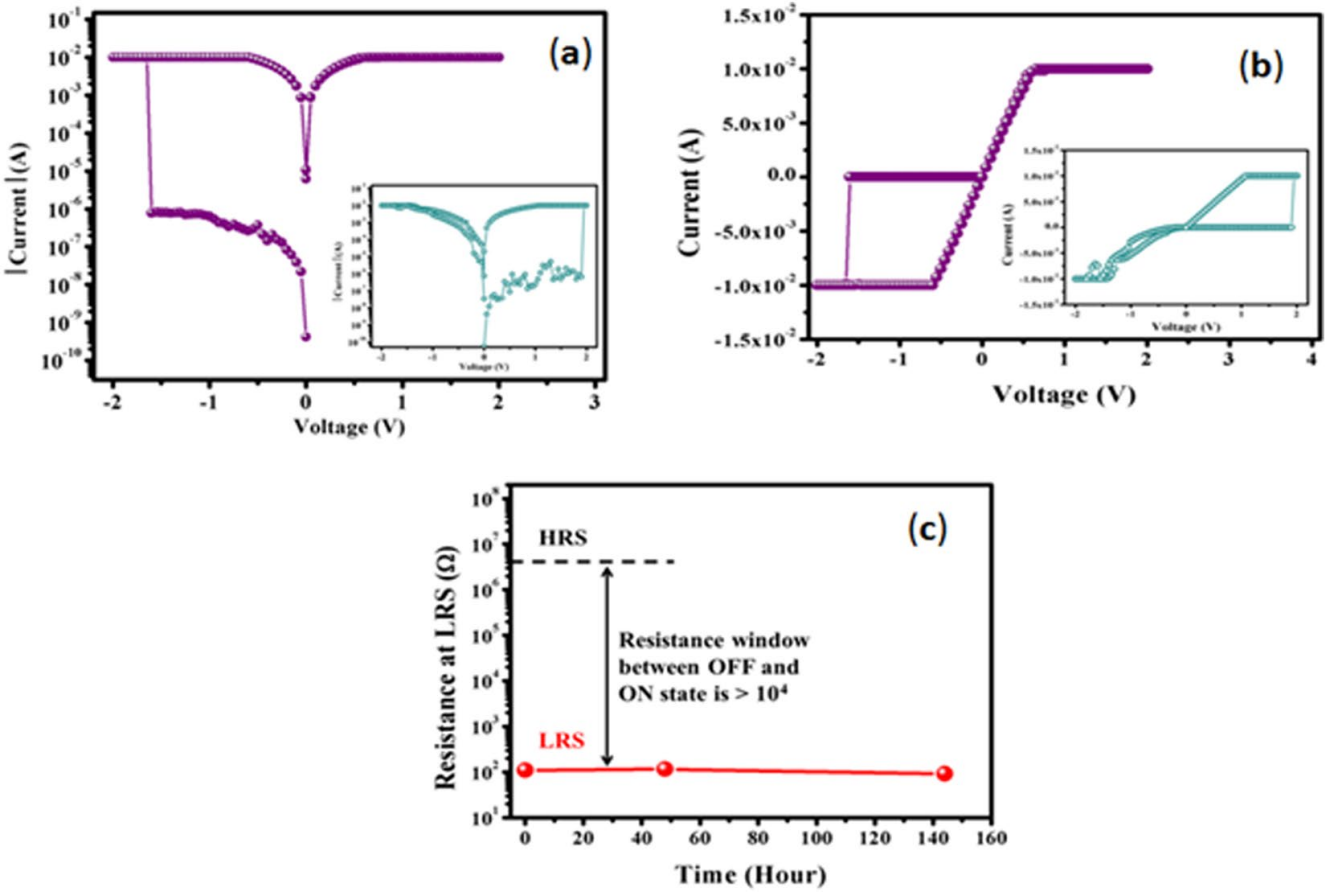
I-V characteristics of the cross-bar memory device comprising ***sample-1*** (**8**:**SA** = 1:3) organic nanomaterials with applied 0 V → −2 V → 0 V →  + 2 V → 0 V and 0 V →  + 2 V → 0 V → −2 V → 0 V (inset) at: (**a**) Semi-log scale and (**b**) Linear scale. (**c**) Retention characteristics of WORM type memory cell.

Current-voltage (I-V) characteristics in semi-log and linear scales for the device consisting of ***sample-1*** organic material are shown in Fig. 4a and Fig. 4b, respectively. As can be observed from the I-V characteristics (Fig. 4a), when the 0 V → −2 V → 0 V →  + 2 V → 0 V voltage sweep sequence was applied, the devices were found to switch from the high resistance state (HRS) to the low resistance state (LRS) at V_TE_ = −1.6 V during the negative sweep; however, the reverse switching from LRS to HRS was not observed when the polarity of the voltage sweep was reversed. A reverse sweep sequence of 0 V →  + 2 V → 0 V → −2 V → 0 V was also applied to another device to check the dependence of the switching behavior on the polarity of the first applied voltage sweep (inset of Fig. 4a and Fig. 4b). The sweep also revealed, from the inset of Fig. 4a and Fig. 4b, that the device indeed switched from HRS to LRS during the positive part of the voltage sweep, whereas switching back from LRS to HRS was not observed during the negative sweep. Once the devices are set to LRS, they do not switch back to HRS, even after the application of large positive (+5 V) and negative (−5 V) voltages. As only unidirectional switching from HRS to LRS is observed when either a positive or negative sweep was applied first, the resistance states of these devices were electrically irreversible. Thus, information once written in this type of memory device cannot be reprogrammed but can be read for a long period of time. Hence, the memory devices are categorized as WORM type, which is suitable for non-editable database, archival memory, electronic voting, and radio frequency identification applications. To validate the application of the device for permanent data storage, a data retention test of these WORM type memory devices was performed. First, the resistance at HRS of one such device was measured at 500 mV; next, the information was written in the same device by means of switching its state from HRS to LRS by applying a positive DC voltage sweep, and then, the stored information was read by measuring its resistance at LRS also at a DC voltage of 500 mV. Because the resistance at LRS, as plotted as a function of time in Fig. 4c, remains almost unchanged after 6 days, this ensures the potential applicability of the device as long term WORM memory.

### *Sample-2*: RRAM characteristics

**Figure 5:**
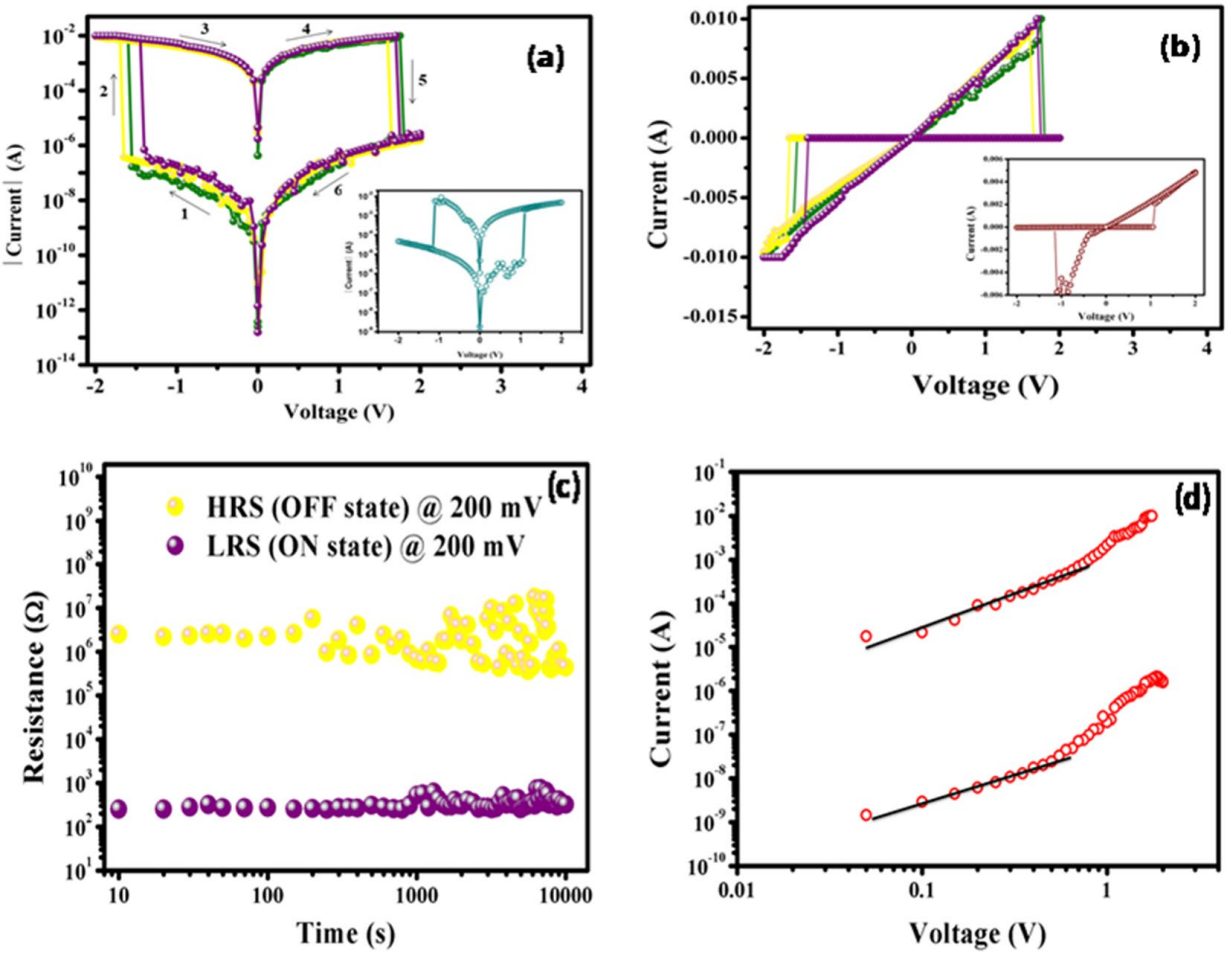
I-V characteristics of the cross-bar memory device comprising ***sample-2*** (**8**:**SA** = 3:1) organic nanomaterials with applied 0 V → −2V → 0 V →  + 2 V → 0 V and 0 V →  + 2 V → 0 V → −2V → 0 V (inset) at: (**a**) Semi-log scale and (**b**) Linear scale. (**c**) Retention characteristics of RRAM device. (**d**) I-V characteristics in log-log scale for the positive cycle of the RRAM device.

After confirming the composition-dependent unique memory application, we were very curious to determine the nature of the I-V characteristics of organic materials made of mixed **8** and **SA** with the reverse order composition, i.e., **8**:**SA** = 3:1 (***sample 2***). We made the device according to the design Fig. 3a with ***sample 2*** and investigated its electrical characteristics. I-V characteristics in both semi-log and linear scales for the devices that used ***sample-2*** as the resistive switching material are shown in Fig. 5. Unlike the devices composed of ***sample-1***, which exhibit unidirectional switching, electrically reversible or bipolar resistive switching was demonstrated in the cross-bar memory devices with ***sample-2*** organic material. When the 0 V → −2 V → 0 V →  + 2 V → 0 V voltage sweep sequence was applied, the device was found to switch from HRS to LRS during the negative sweep, and subsequently, the positive part of the voltage sweep cycle switched the device back to HRS from LRS. Conversely, a reverse sweep sequence, 0 V →  + 2 V → 0 V → −2 V → 0 V, when applied to another device, also switched the device from HRS to LRS and back from LRS to HRS during positive and negative parts of the voltage sweep, respectively. As illustrated in Fig. 5a, when the voltage was swept from 0 V to −2 V, as the resistance of the material was initially high, the memory device remains OFF (Step 1) until it is set to ON at a certain voltage (V_set_), when the material abruptly changes its state from HRS to LRS (Step 2). The device remained ON during the period when the voltage was swept back from −2 V to 0 V (Step 3). Even when a positive voltage sweep was applied (Step 4), the device could maintain the ON state up to a certain voltage (V_reset_), at which point the device was reset to OFF as the material proceeded through another abrupt change in resistance from LRS to HRS (Step 5). The OFF state was maintained when the voltage was scanned back from 2 V to 0 V (Step 6). Although, the set voltage V_set_ and the reset voltage V_reset_ were found to vary for repeated switching cycles, the maximum value of V_set_ = −1.3 V and minimum value of V_reset_ = 1.4 V were observed in our experiment (Fig. 5b). The bipolar nature of ***sample-2*** ensures electrical reversibility of its resistance state between HRS and LRS, maintaining a sufficiently large resistance window. Hence, the cross-bar memory devices utilizing ***sample-2*** are suitable for RRAM applications.

The data retention performance of the RRAM device under ambient conditions is presented in Fig. 5c. For the study of the retention time at HRS, one of the memory devices was first switched from HRS to LRS by applying a negative (−2 V) voltage sweep and then switched back from LRS to HRS by applying a positive (+2 V) DC voltage sweep. Similarly, a retention study at LRS was performed on another device, which was sequentially switched from HRS to LRS, followed by LRS to HRS and then again from HRS to LRS by applying negative (−2 V), positive (+2 V) and negative (−2 V) dc voltage sweeps. Both the resistances at LRS and HRS were continuously measured at 200 mV for more than 10^4^ seconds. Figure 5c reveals that even after 10^4^ seconds, the device maintained a resistance memory window greater than 10^2^ Ohms, which is sufficient for RRAM applications. Further insight regarding the conduction mechanism of the RRAM device may be found from Fig. 5d, which shows a plot of the I-V characteristics for the positive cycle in the log-log scale. The current at both LRS and HRS followed a linear relation, which indicates Ohmic conduction behavior at low voltages. However, for higher voltages, the current in both states exhibited quadratic dependence with voltage, which might be due to shallow trap assisted space charge limited conduction^14^.

### Plausible polarizing mechanism of switching at the molecular stage

**Figure 6:**
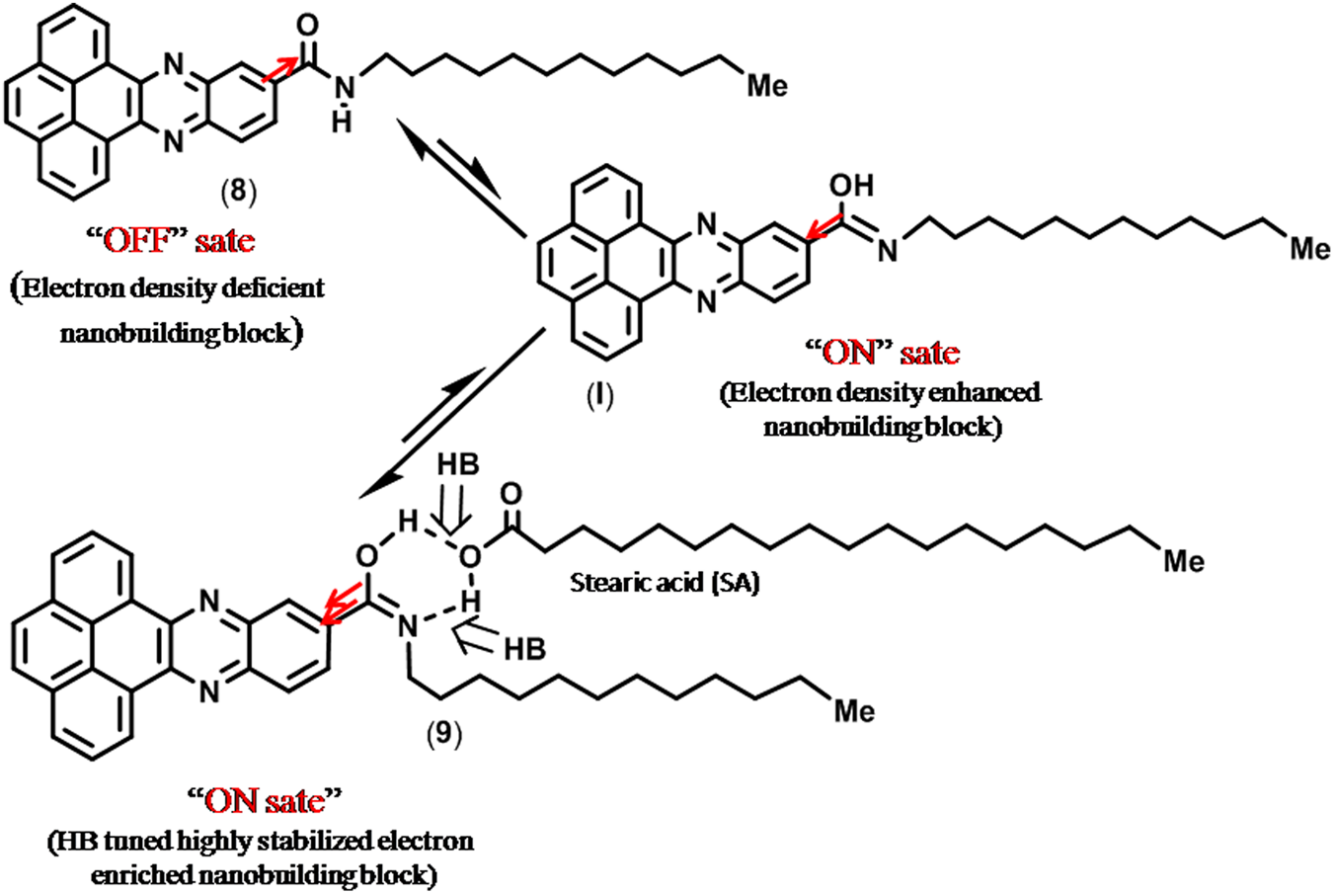
Possible composition dependent polarization of chemical structure of **8** with voltage and in the presence of acid (**SA**).

A number of mechanisms for the two switching states have been proposed, such as conformational changes, rotation of the functional group, charge transfer, reduction–oxidation processes, filamentary conduction, space charges and traps, and ionic conduction^49,50^. Herein, the most reasonable mechanism for the appearance of smooth RS activity of compound **8** is due to the presence of the amide electron withdrawing group (C=O), highly electron rich aromatic nucleus, and (above all) unidirectional packing of the nanobuilding blocks in the organic nanomaterials for easy charge transport. The applied forward bias opposes the acceptor group for the non-conjugation, i.e., perturbation in conjugation will be reduced and the localized state must dominate the carrier transport whenever conformational switching occurs. As a result, the acceptor group (C=O) is transformed into the –OH group and C–NH becomes C=N. During scanning at lower voltage, we initially observed a low conducting state (OFF state) in the typical I-V characteristic of **8** in the cross bar device. Herein, we expect that the switching behavior of **8** is due to the presence of the electron-acceptor (C=O) group of amide, and this functionality might perturb the conjugation with significant reduction of the π-electron clouds over the surface of nanobuilding block **8**, resulting in the low current state (OFF). When the voltage approaches the so-called threshold voltage (~2 V), the current increases rapidly, showing its high conducting state (ON state). During enhancement of the applied biased voltage across the device, the device switches to the ON state because of the electric field induced structural rearrangement (**I**, Fig. 6), and ***sample 2*** was quite stable in the ON state because of the presence of a small amount of stabilizing acid. Interestingly, in the case of ***sample 1*** nanomaterials, the device stabilizes through strong hydrogen bonding caused by the presence of a large excess amount of acid (**SA**, Fig. 6), i.e., the complete absence of electron acceptor groups in the structure; in addition, there arises a stable electron donating group (C=N) in the molecular structure without the field induced conformational changes. As a result, the electron distribution in the phenazine moiety rearranges through polarization, and hence, conjugation of the molecule is restored, resulting in an overlapping electronic structure. Expectantly, herein, switching occurred (Fig. 4) earlier than for ***sample 2*** (Fig. 5). The behavior of composition-dependent innovative WORM and RRAM organic electronics of the cross bar device (Fig. 3a) may be explained by the different packing patterns in the nanoscale materials, which ultimately generated two completely different nanoelectronic devices for two useful memory applications.

As the electron-accepting groups behave as the “Charge traps”, mobility of the charge carriers in the compound **8** is low due to the presence of electron-acceptor (C=O) group^30^ of amide. For the devices with ***sample 2***, initially for low scanning voltage when the carrier injection from the contact is weak, conductivity of the material is low as most of the electrons are trapped by the electron-accepting group. The material maintains the low conductivity state till the so called threshold voltage is reached. Gradual increase of scanning voltage gradually enhances the carrier injection level that fills more and more number of traps. Eventually, a condition will arrive when all of the traps are filled with injected electrons and further increase of scanning voltage drives the material to switch its conductivity from low to high. The material maintains the high conductivity when the bias voltage is scanned back to zero. During the other half of this voltage sweep cycle, as the voltage polarity is made opposite, an opposite mechanism will occur, i.e., a condition will reach when no traps will remain filled and beyond that point the material switch back from high conductivity to low conductivity state. It can be seen from Fig. 2f that the nanomaterials in ***sample 2*** are closely packed that eases the carrier transports from one molecule to the next neighbor molecule. We expect that such intermolecular transport favors the bidirectional conductivity switching. On the other hand, as the ***sample 1*** contains large amount of SA, the distribution of nanomaterials are scattered. It can be visualized from the TEM image of ***sample 1*** (Supplementary Infoemation) that the average distance between one nanomaterial to the nearest neighbor is >100 nm. Therefore, one may expect that charge transport for ***sample 1*** is dominated by carrier hopping^31^. Also for this material, conductivity switching may occur due to the filling of traps of all of the individual nanomaterials. Unlike the memory device consists of ***sample 2***, once the device with ***sample 1*** is switched to high conductivity state, reverse switching is unfavorable for this device because of such scattered arrangement of nanomaterials. It is to be noted that overall electronic sensitivity and characteristics of the fabricated devices are expected to be much different in comparison to the same for the individual nanobuilding blocks (**8**, **I**, **9**).

## Discussion

In contrast to a small number of reported methods and their limitations, we demonstrated a new metal-free sustainable strategy in this research article to directly obtain designed phenazine compounds bearing strong π-electrons, voltage-dependent electron withdrawing and donating amide functionality, chirality and long chain hydrocarbons through multicomponent cyclization with amidation at ambient temperature (Fig. 1). The new phenazine compounds were designed by density functional theory (DFT) as implemented in Gaussian 09 software (Supplementary Information) and found to be efficient nanobuilding blocks due to the presence of several noncovalent attractive forces, such as hydrogen bonding, π−π stacking, van der Waals and induced dipole interactions, which were efficiently used for the fabrication of several uniformly packing 1D materials for *de novo* organic nanoelectronics. We investigated the organic monolayer characteristics of pure phenazine (**8**) and mixed film behavior with possible binding interactions using a long chain fatty acid **SA** at the air-water interface and assembly onto a solid support using the LB technique. Our measurements on the thermodynamic behavior of pure and mixed films provided important features of the mixed materials, e.g., there were strong interactions between **8** and **SA** in the solid state and the maximum interaction occurred for approximately the 1:3 mole ratio (**8**:**SA**), at which the most stable monolayer was achieved. The AFM imaging of a pure LB monolayer film of **8** exhibited few circularly nanodisk-like structures, which are merely the cross sections of the nanofibrils of **8** (SEM images, Supplementary Information). The porous-like structure of the **8**-**SA** LB monolayer film was an outstanding observation from the application point of view to achieve futuristic high-performance nanoscale devices. The most striking feature of the newly synthesized amido-phenazine **8** is that it revealed two different types of innovative composition-dependent resistive switching properties: a mixture of **8** with **SA** (3:1) can be switched repeatedly between HRS and LRS by applying alternate polarity of the voltage sweep or pulse and is suitable for ultra-fast RRAM application; a mixture of **8** with **SA** in the reverse ratio (1:3) can be switched only once in a unidirectional fashion from HRS to LRS, making it ideal for WORM memory. Ideally, the memory device should maintain stable state in both the on and off states. In this case, the memory retention performance was quite good up to 1000 s, after which the fluctuation of resistance in the off state was found to be higher. Such performance degradation is, however, very common as reported by Khurana^51^ and Cho *et al*.^52^ in their publications. Also, Fig. 5c revealed that even after 10^4^ seconds, the device maintained orders of magnitude tail-to-tail resistance gap or memory window that is sufficient for RRAM applications.

In contrast to inorganic materials, memory devices are insensitive to moisture and are less sensitive to magnetic interference; the unique I-V characteristics were explained by introducing a new polarization mechanism. The novel composition dependent WORM-RRAM organic nanoelectronics of amido-phenazine material-based cross bar devices will find considerable application in electronic device industry, and this work simultaneously opens a path for intensive future investigations to address a variety of key issues. First, an extensive investigation on device processing condition may result in the discovery two different types of switching resistors. Second, the chemical structure and organic material dependent study may achieve nanoelectronic devices for *de novo* switching characteristics. Third, once the processing and structural differences are known, it would then be interesting to establish the electronic mechanism responsible for the two different types of switching characteristics. Fourth, intensive research will be conducted for immediate commercial uses of the innovative organic nanomaterials in high-tech device application.

## Electronic supplementary material

Supplementary Information
