# Supplementary material for: Composition-dependent nanoelectronics of amido-phenazines: non-volatile RRAM and WORM memory devices
Source: Sci Rep. 2017 Oct 17;7:13308. doi: 10.1038/s41598-017-13754-w (PMC5645374; doi:10.1038/s41598-017-13754-w)
Supplement: Supplementary file 1 — Supplementary Information [file 41598_2017_13754_MOESM1_ESM.doc]

**Supplementary Information**

**Composition-dependent nanoelectronics of amido-phenazines: non-volatile RRAM and WORM memory devices**

**Dilip K. Maiti,^§^* Sudipto Debnath,^§^ Sk. Masum Nawaz,^¶^ Bapi Dey,^ɠ^ , Enakhi Dinda,^§^ Dipanwita Roy,^§^ Sudipta Ray,^§^ Abhijit Mallik,^¶^* and Syed A. Hussain^ɠ^***

**^§^Department of Chemistry, University of Calcutta, 92 A. P. C. Road, Kolkata-700009, India, e-mail: dkmchem@caluniv.ac.in**

**^¶^Department of Electronic Science, 92 A. P. C. Road, Kolkata-700009, India, e-mail: amelc@caluniv.ac.in**

**^ɠ^Department of Physics, Tripura University, Suryamaninagar -799022, Tripura, India, e-mail:**

***Corresponding author. Fax: 91-33-2351 9755, Tel: 91-33-2350 1014**

Serial No. Content Page Numbers

1. Materials and methods S-2

2. Synthesis of pyrene-4,5-dione (**1**) and its characterization data S-3

3. General procedure for the synthesis of phenazine compounds (**4**-**8**) S-4

4. Characterization data of the synthesized phenazine compounds (**4-8**) S-4

5. ^1^H and ^13^C NMR spectra of all new phenazine compounds (**4-8**) S-7

6. Design and packing of phenazine nanobuilding block (**8**) with Z-matrix S-12

7. Morphology of organic nanomaterials of pure **4-8** and mixed **8+SA** S-20

8. Isotherm measurement and film formation S-21

9. BAM experiments S-22

10. UV-Vis study S-24

11. References S-24

**1. Materials and methods**

All solvents were dried by standard methods. Unless otherwise specified, chemicals were purchased from commercial suppliers and used without further purification. Column chromatography was performed on silica gel (60-120 mesh). TLC was done on glass sheets pre-coated with silica gel (with binder, 300 meshes, Merck). Synthetic procedures, characterization data and NMR spectra of new compounds **2**-**8** were documented. The ^1^H- and ^13^C-NMR spectra were taken in CDCl_3_ with TMS as an internal standard on Bruker Supercon NMR spectrometer (Model: AV 300 Digital). The chemical shifts were reported as δ values (ppm) relative to tetramethylsilane. Proton multiplicities are represented as s (singlet), d (doublet), dd (double doublet), t (triplet), q (quartet), and m (multiplet). Infrared spectra were recorded on FTIR spectrometer in KBr pellets and in NaCl cell (liquid sample) on a Perkin-Elmer RXI-FTIR spectrophotometer. Melting points of the samples were determined with a Fisher-John melting point apparatus and were uncorrected. HR-MS data were acquired by electron spray ionization technique on a Q-tof-micro quadriple mass spectrophotometer (Waters XEVO G2-S QTof).

Fabrication of organic nanomaterials was performed in a spin coater (Apex Instruments Co., SPU 2005A) under vacuum using DMF as a solvent. A Langmuir-Blodgett (LB) film deposition instrument (Apex 2000C, Apex Instruments Co.) was used for surface pressure–area isotherm measurements and monolayer film preparation. Ultra pure Milli-Q water of resistivity 18.2 MΩ–cm was used as sub-phase. Morphology of the fabricated nanomaterials of pure and mixed phenazines was determined in the scanning electron microscope (SEM, Carl Zeiss) and FEG-SEM (JEOL). Scanning transmission electron microscopy (TEM) images and electron energy loss spectrum (EELS) were taken using an ultra-high resolution field emission gun transmission electron microscope (UHR-FEG TEM, JEM-2100F, JEOL) operating at 200 kV. UV-Vis absorption and fluorescence of pure solutions and those of mixed LB films were recorded using absorption spectrophotometer (UV-Vis-NIR, Perkin Elmer, Lambda 750) and fluorescence spectrophotometer (Perkin Elmer, LS 55) respectively. The absorption spectra were recorded at 90^0^ incidence and using a clean quartz slide as reference. The current–voltage (I–V) characteristics were measured by using Keithley 2401 source meter. The AFM image of monolayer film was taken with a commercial AFM system, Innova AFM system (Bruker AXS Pte Ltd.) by using silicon cantilevers with a sharp, high apex ratio tip (Veeco Instruments). The AFM images presented here was obtained in intermittent-contact (“tapping”) mode. The excess Gibbs free energy of mixing ($\Delta G_{exc}$) for the mixed monolayer system has been calculated using software MATLAB 9.0.

**2. Synthesis of pyrene-4,5-dione (1) and its characterization data**

**SI** **Figure 1│** Ru(III) catalyzed periodate oxidation of pyrene

Pyrene (2.0 mmol, 404.52 mg) was taken in a mixture of solvents of dichloromethane (8 mL), acetonitrile (8 mL) and water (8 mL). NaIO_4_ (9.36 mmol, 2 gm) and catalytic amount of RuCl_3_.xH_2_O (0.192 mmol, 40 mg) were added to the reaction mixture at room temperature (rt). The reaction mixture was allowed to stir at rt for 18 h. The reaction was monitored by thin layer chromatography (TLC). The post-reaction mixture was filtered through cellite and filtrate was extracted with EtOAc (2x15 mL), and the combined organic layer was washed with water (3x10 mL) and brine (1x10 mL). It was dried over anhydrous Na_2_SO_4_, filtered and evaporated in a rotary evaporator under reduced pressure at room temperature. The crude product was purified by column chromatography on silica gel (60-120 mesh) with ethyl acetate-petroleum ether (20%, v/v) as an eluent, which afforded the corresponding pyrene-4,5-dione (**1**) in 70% yield.

**Characterization data of compound 1**

**Pyrene-4,5-dione (1)**

**Yield:** 70% (162.4 mg, 0.7 mmol).

**Characteristic:** Yellow solid.

**M.P.** 190-191 ^o^C

**^1^H NMR** (300 MHz, CDCl_3_): δ 7.28 (2H, s), 7.74-7.79 (2H, m), 8.17-8.20 (2H, m), 8.48-8.51 (2H, m).

**^13^C NMR** (75 MHz, CDCl_3_): δ 121.5, 124.2, 128.8, 128.8, 131.2, 131.2, 137.4, 137.5, 189.1.

**FT-IR** (KBr, cm^-1^): 1079, 1180, 1409, 1535, 1640, 2932.

**HRMS** (ESI-TOF) m/z Calcd for C_16_H_9_O_2_ [M+H]: Calculated 233.0603, found 233.0608.

**3. General procedure for the synthesis of phenazine compounds (4-8)**

**SI** **Figure 2│**Metal-free oxidative heterocyclization with installation of amide functionality

Aromatic-1,2-diketones (**1**, 1 mmol) and 3,4-diamino carboxylic acid (**2**, 1 mmol) were dissolved in dry DMSO under argon atmosphere at ambient temperature, and stirred for 2 h untill disappearence of the starting material was observed by TLC. CS_2_ (1.2 mmol) and PhIO (1.5 mmol) were added to the reaction mixture and allowed to stir for 30 min. The long chain amine compound (**3**, 1.1 mmol) was added to the reaction mixture and allowed to stirr at rt for 12 h. Progress of the reaction was monitored by TLC. Ethylacetate (50 mL) was added to the post-reaction mixture and was washed with ice water for 7-8 times to remove the traces of DMSO from the reaction mixture. The organic layer was washed with brine (1x10 mL). It was dried over anhydrous Na_2_SO_4_, filtered and evaporated in a rotary evaporator under reduced pressure at rt. The crude product was purified by column chromatography on silica gel (60-120 mesh) with ethyl acetate-petroleum ether (15%, v/v) as an eluent, which afforded the corresponding long chain amide **4**, **5** and **8**. Same procedure was followed for synthesis of compound **6** and **7**, only difference was the ratio of acid and amine utilized in the reaction. In case of compound **6** acid-long chain amine ratio was 2:1 *i.e* 1 mmol of acid and 0.5 mmol diamine, and for compound **7**, 1 mmol acid, 0.35 mmol *tris*-amine were utilized.

**4. Characterization data of the synthesized phenazine compounds (4-8)**

**4.1. *N*-Dodecylbenzo[*a,c*]phenazine-11-carboxamide (4)**

**Yield:** 75% (369 mg, 0.75 mmol).

**Characteristic:** Yellow solid.

**M.P.** 204-205 ^o^C

**^1^H NMR** (300 MHz, CDCl_3_): δ 0.85 (3H, t, *J* = 7.2 Hz), 1.24-1.36 (18H, m), 1.64-1.71 (2H, m), 3.52-3.58 (2H, m), 7.71-7.83 (4H, m), 8.25-8.39 (2H, m), 8.53-8.63 (2H, m), 8.64 (1H, s), 9.35-9.40 (2H, m).

**^13^C NMR** (75 MHz, CDCl_3_): δ 14.1, 22.7, 27.1, 29.3, 29.6, 29.6, 29.7, 29.7, 32.0, 40.5, 123.1, 126.6, 128.1, 128.3, 130.0, 130.8, 130.9, 132.3, 132.4, 141.2, 143.3, 143.6, 166.7.

**FT-IR** (KBr, cm^-1^): 756, 1027, 1090, 1204, 1258, 1363, 1449, 1534, 1629, 1714, 2361, 2852, 2921, 3433

**HRMS** (ESI-MS) m/z Calcd for C_33_H_38_N_3_O [M+H]: Calculated 492.3015, found 492.3018.

**4.2. *N*-Octadecyldibenzo[*a,c*]phenazine-11-carboxamide (5)**

**Yield:** 81% (465.25 mg, 0.81 mmol).

**Characteristic:** Yellow solid.

**M.P.** 210-212 ^o^C

**^1^H NMR** (300 MHz, CDCl_3_): δ 0.80 (3H, t, *J* = 6.6 Hz), 1.15-1.21 (30H, m), 1.78-1.80 (2H, m), 3.41-3.50 (2H, m), 7.66-7.78 (4H, m), 8.27-8.38 (2H, m), 8.48-8.57 (2H, m), 8.98 (1H, s), 9.31-9.35 (2H, m).

**^13^C NMR** (75 MHz, CDCl_3_): δ 14.1, 22.5, 26.9, 29.2, 29.4, 29.5, 29.7, 29.8, 34.6, 42.9, 123.1, 126.5, 128.0, 128.1, 128.8, 130.7, 130.9, 132.3, 132.4, 140.9, 143.3, 143.4, 166.6.

**FT-IR** (KBr, cm^-1^): 759, 1030, 1098, 1200, 1260, 1370, 1455, 1539, 1637, 1719, 2368, 2857, 2929, 3438

**HRMS** (ESI-TOF) m/z Calcd for C_39_H_50_N_3_O [M+H]: Calculated 576.3954, found 576.3980.

**4.3. N,N'-((1R,2R)-Cyclohexane-1,2-diyl)didibenzo[*a,c*]phenazine-11-carboxamide (6)**

**Yield:** 80% (580 mg, 0.8 mmol).

**Characteristic:** Pale yellow oil.

**M.P.** 289-2910 ^o^C

**[α]_D_^20^** = -13.9° (c 1.2, CHCl_3_)

**^1^H NMR** (300 MHz, CDCl_3_): δ 1.12-1.26 (4H, m), 1.41-1.50 (4H, m), 3.98-4.50 (2H, m), 7.73-7.84 (8H, m), 7.90-7.94 (4H, m), 8.36-8.39 (4H, m), 8.555 (2H, s), 9.37-9.42 (4H, m).

**^13^C NMR** (75 MHz, CDCl_3_): δ 29.3, 35.1, 54.0, 122.4, 125.8, 126.1, 127.4, 127.5, 128.2, 129.4, 129.5, 130.2, 130.3, 131.7, 131.9, 136.7, 140.8, 141.7, 142.7, 142.9, 170.1.

**FT-IR** (KBr, cm^-1^): 1066, 1184, 1360, 1450, 1497, 1541, 1630, 2928, 3455.

**HRMS** (ESI-TOF) m/z Calcd for C_48_H_35_N_6_O_2_ [M+H]: Calculated 727.2821, found 727.2817.

**4.4. N,N',N''-(2,2',2''-Nitrilotris(ethane-2,1-diyl))tridibenzo[*a,c*]phenazine-11-carboxamide (7)**

**Yield:** 65% (691 mg, 0.65 mmol).

**Characteristic:** Yellow solid.

**M.P.** >250^o^C

**^1^H NMR** (300 MHz, CDCl_3_): δ 2.48 (6H, t, *J* = 6.3 Hz), 3.49-3.58 (6H, m), 7.74-7.85 (12H, m), 7.91-7.95 (6H, m), 8.37-8.39 (6H, m), 8.56 (3H, s), 9.36-9.44 (6H, m).

**^13^C NMR** (75 MHz, CDCl_3_): δ 39.3, 57.1, 122.5, 125.9, 126.0, 127.5, 127.6, 128.1, 129.5, 129.68, 130.2, 130.2, 131.8, 131.8, 136.8, 140.9, 141.8, 142.8, 142.8, 170.1.

**FT-IR** (KBr, cm^-1^): 1070, 1190, 1369, 1460, 1490, 1561, 1645, 2967, 3458.

**HRMS** (ESI-TOF) m/z Calcd for C_69_H_49_N_10_O_3_ [M+H]: Calculated 1065.3989, found 1065.3991.

**4.5. *N*-Dodecylphenanthro[4,5-*abc*]phenazine-11-carboxamide (8)**

**Yield:** 77% (397 mg, 0.77 mmol).

**Characteristic:** Pale yellow solid.

**M.P.** 200-202 ^o^C

**^1^H NMR** (300 MHz, CDCl_3_): δ 0.88 (3H, t, *J* = 6.9 Hz), 1.30-1.37 (18H, m), 1.67-1.73 (2H, m), 3.57-3.64 (2H, m), 8.05-8.13 (3H, m), 8.31 (2H, d, *J* = 8.4 Hz), 8.41-8.44 (2H, m), 8.66 (1H, s), 9.53-9.59 (3H, m).

**^13^C NMR** (75 MHz, CDCl_3_): 14.1, 22.7, 27.1, 29.2, 29.4, 29.5, 29.7, 29.7, 31.8, 40.7,112.0,113.5, 123.06, 126.6, 128.1, 128.2, 129.9, 130.7, 130.8, 132.2, 132.4, 136.6, 144.0, 146.2, 146.7, 147.0, 150.9, 170.5.

**FT-IR** (KBr, cm^-1^): 1100, 1232, 1269, 1376, 1430, 1549, 1624, 1729, 2370, 2865, 2954, 3400

**HRMS** (ESI-TOF) m/z Calcd for C_35_H_38_N_3_O [M+H]: Calculated 516.3015, found 516.3014.

**5. ^1^H and ^13^C NMR spectra of all new phenazine compounds (4-8)**


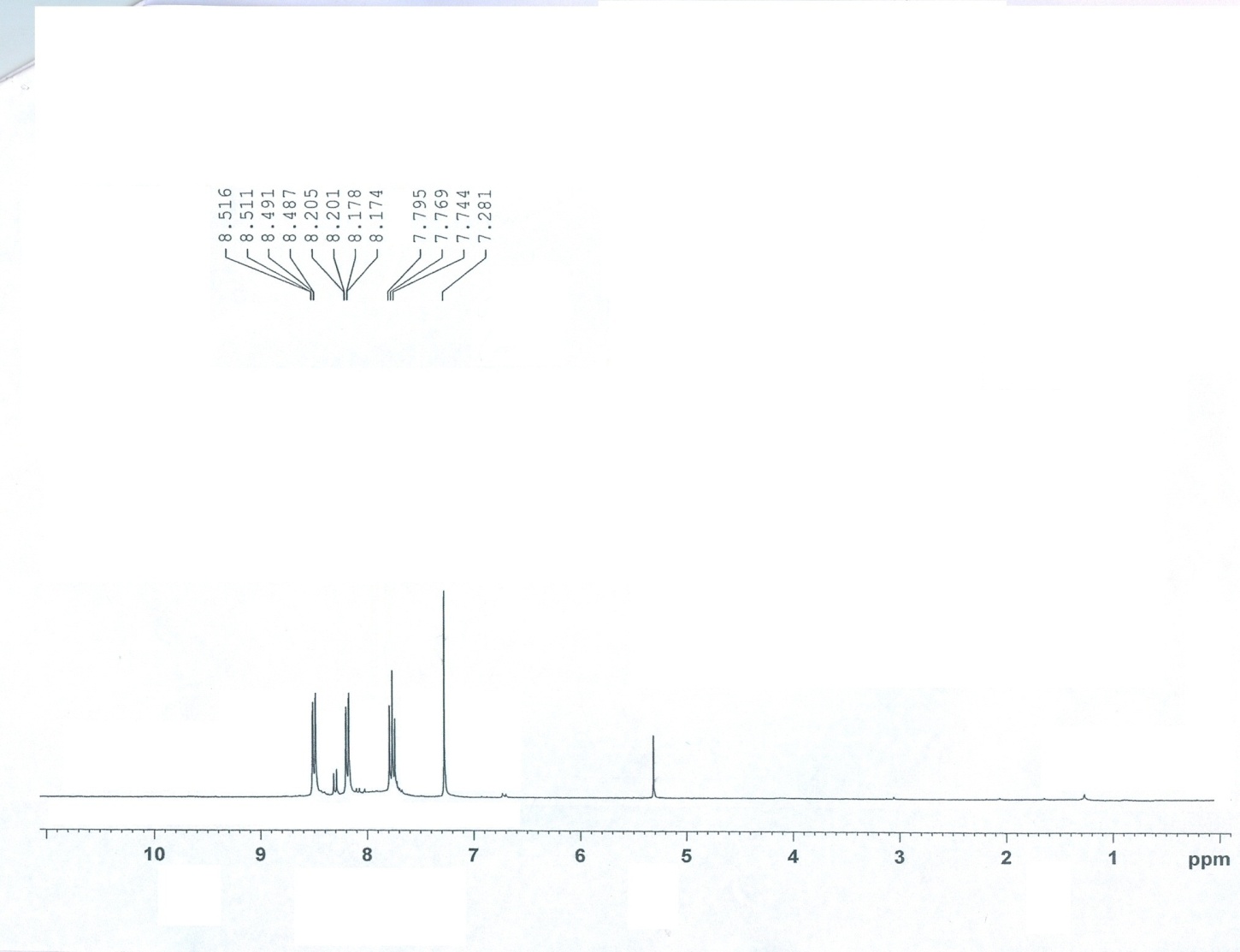


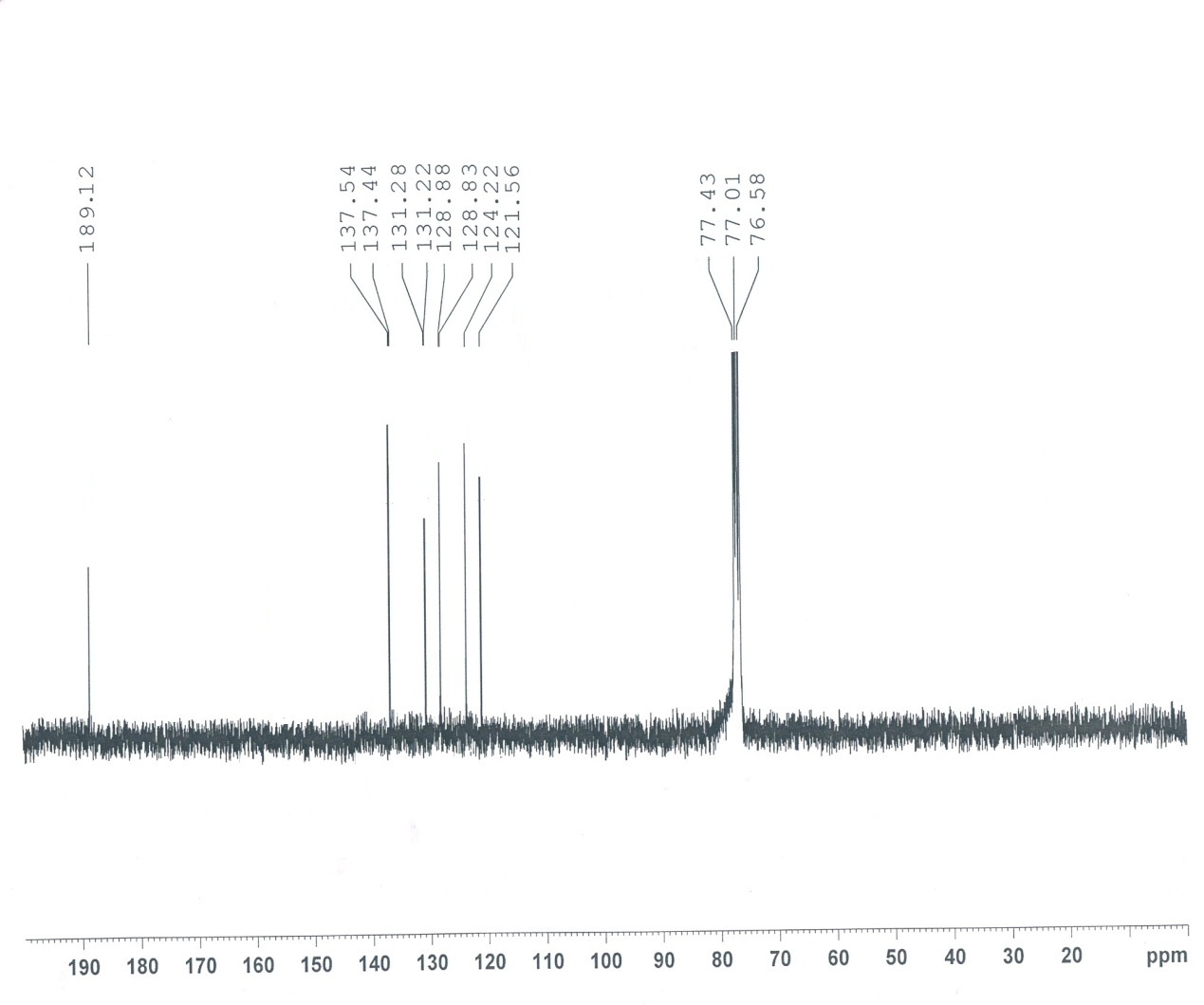


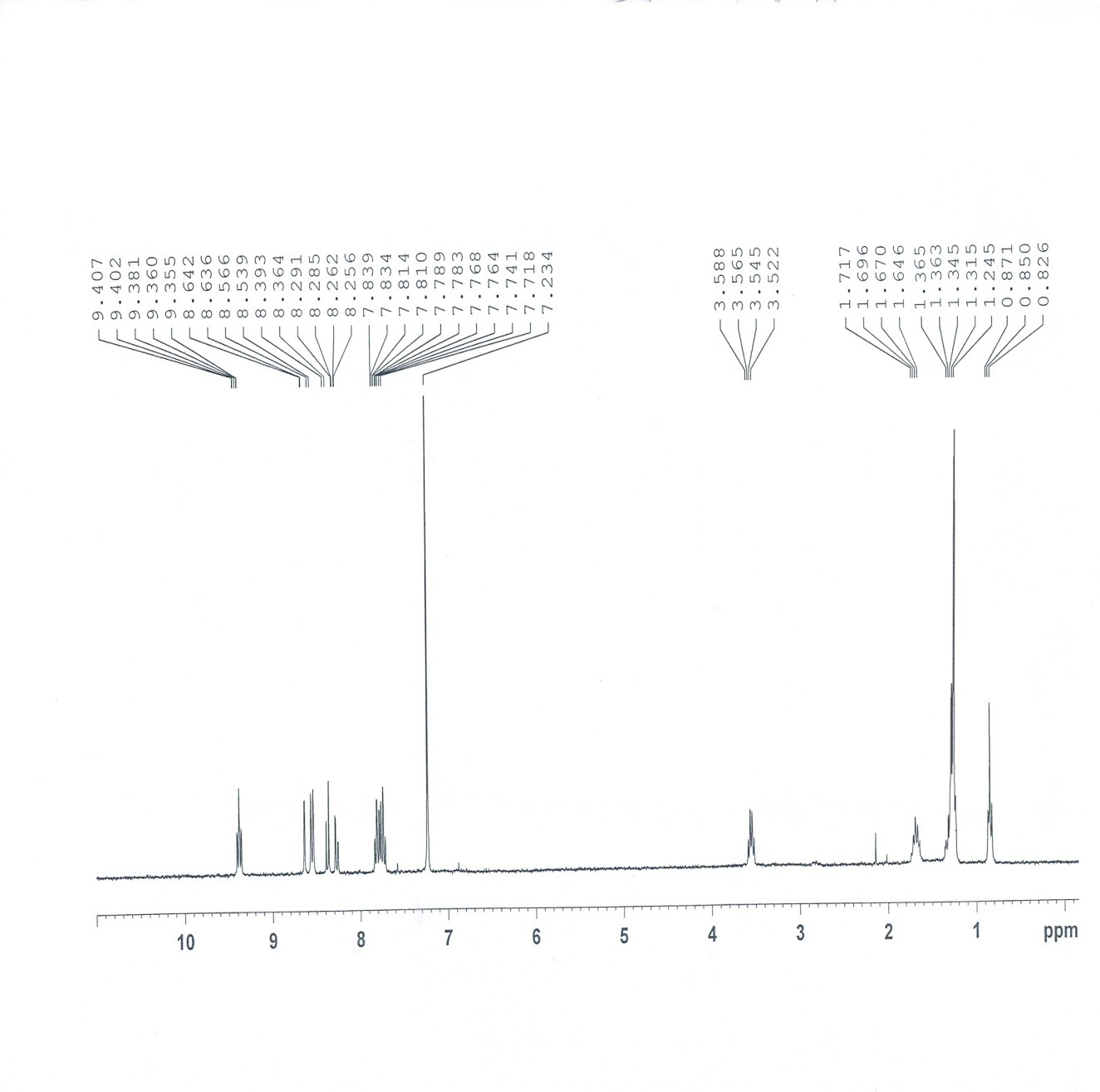


**
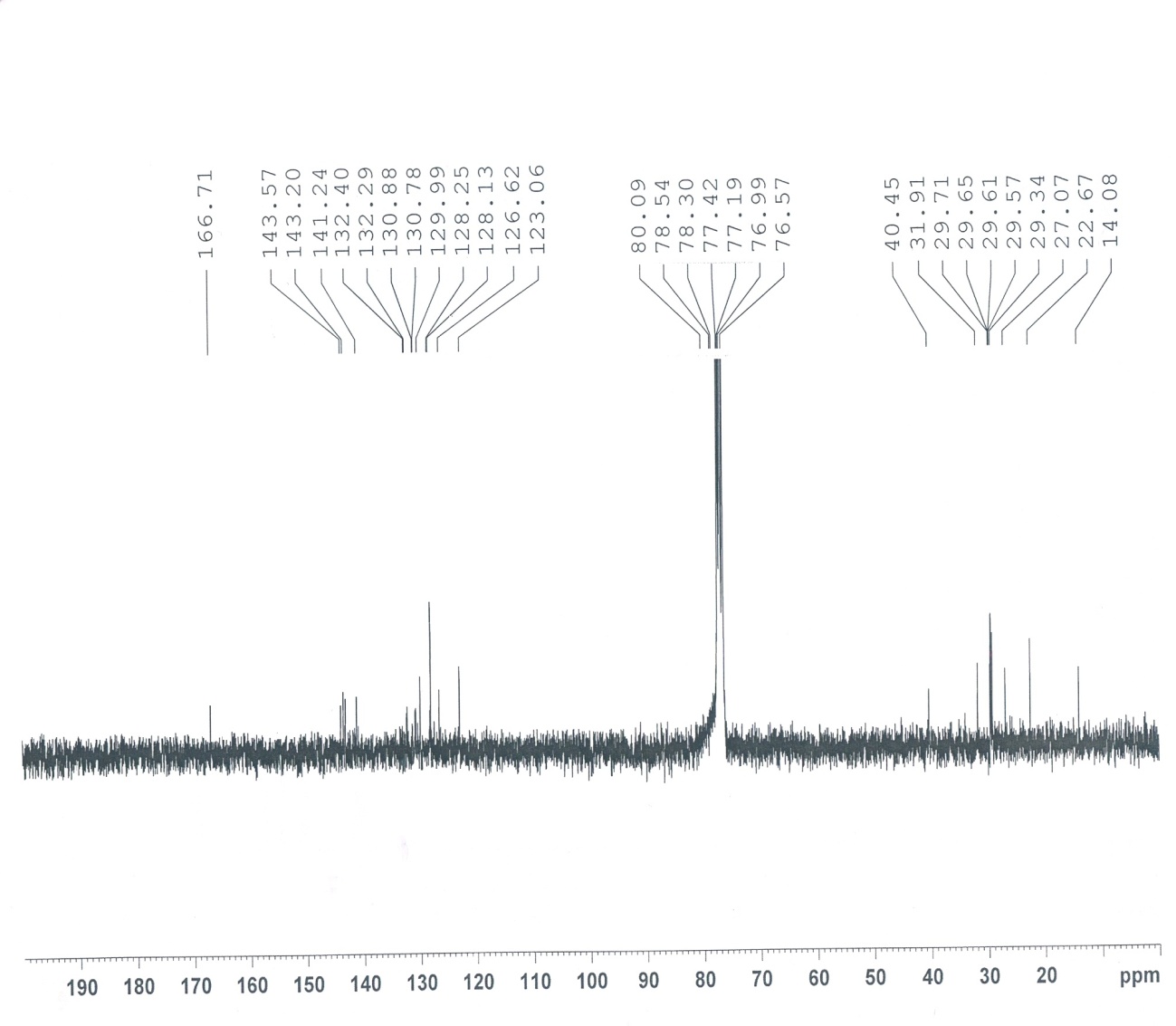
**

**
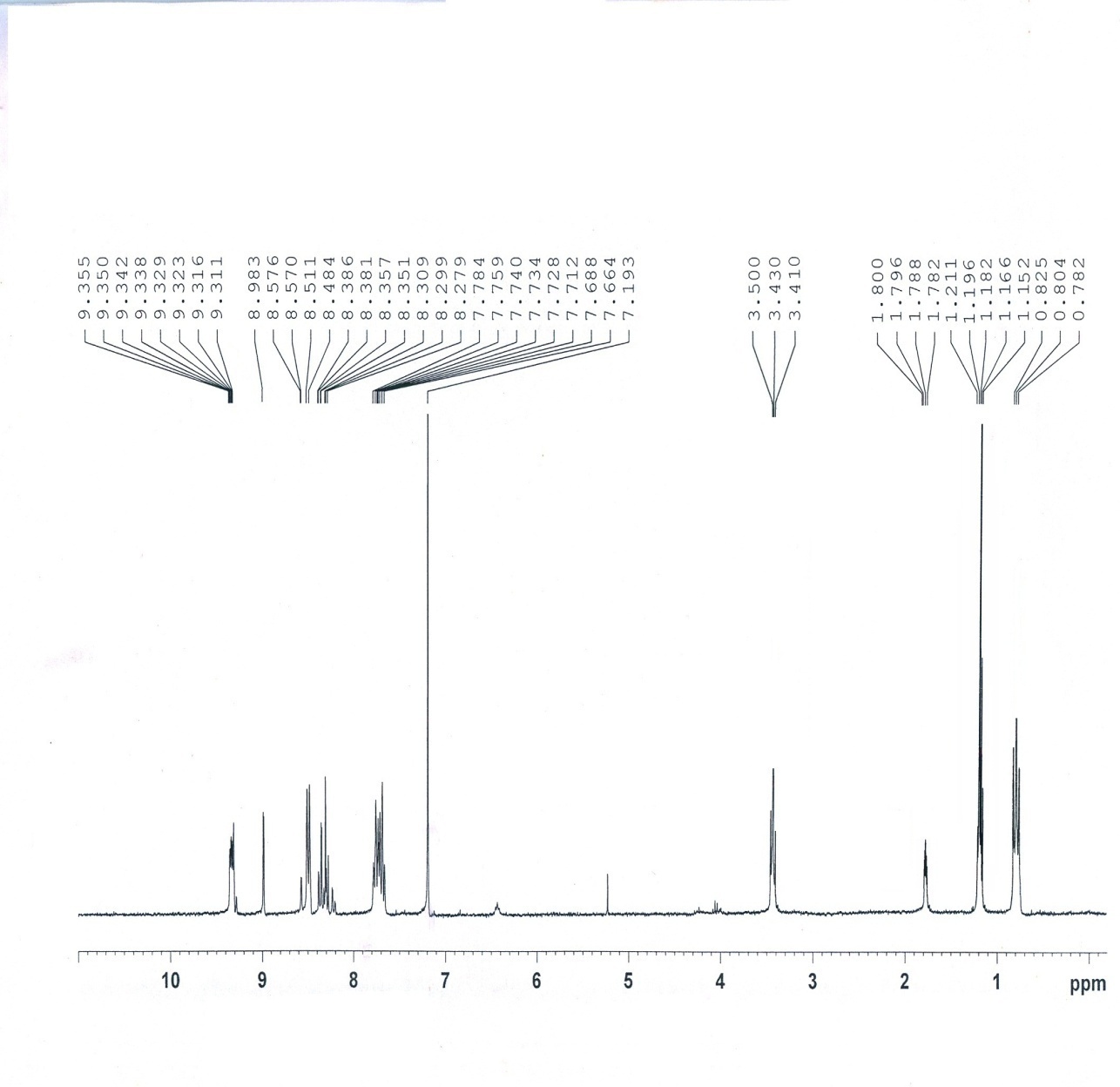
**

**
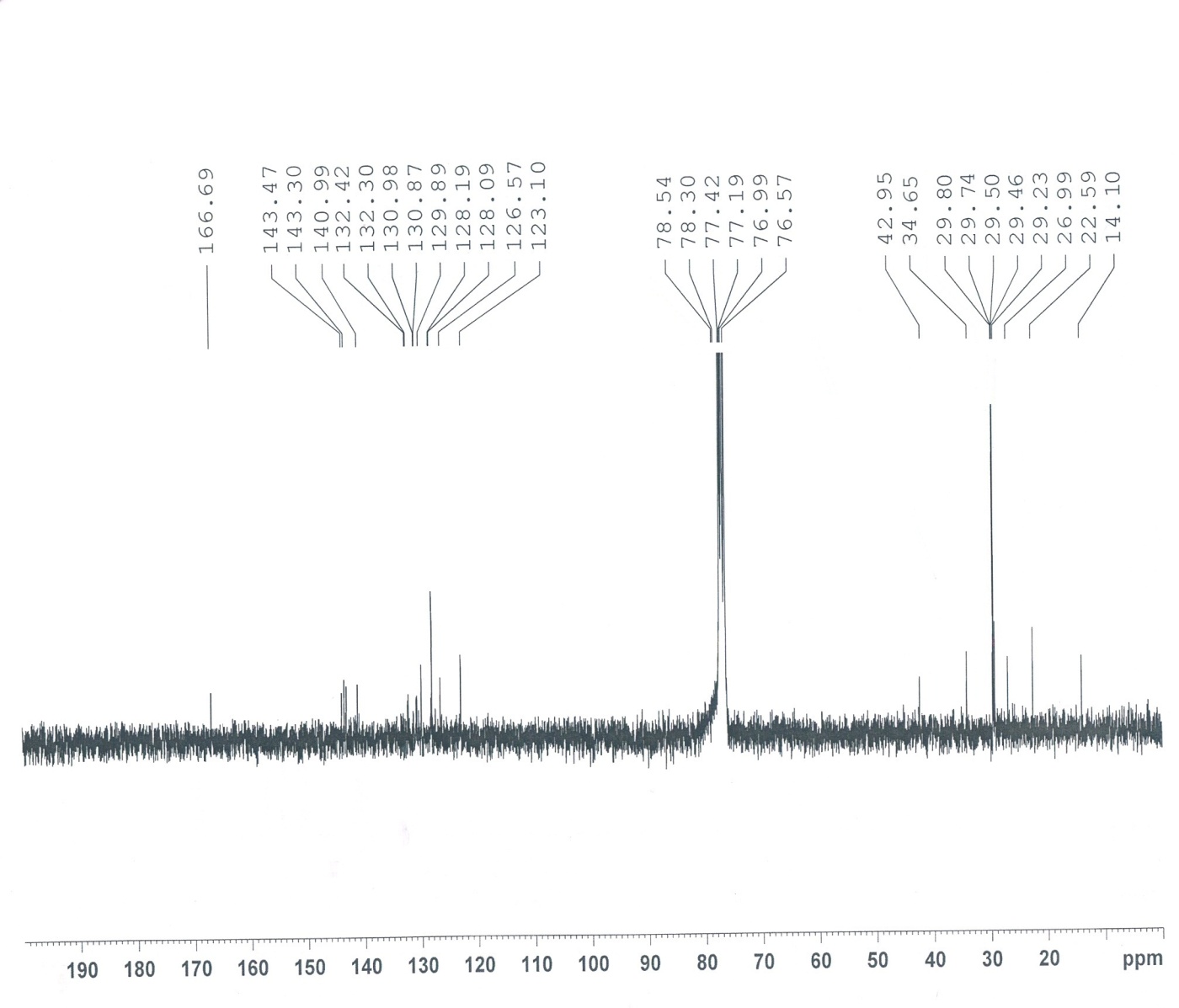
**

**
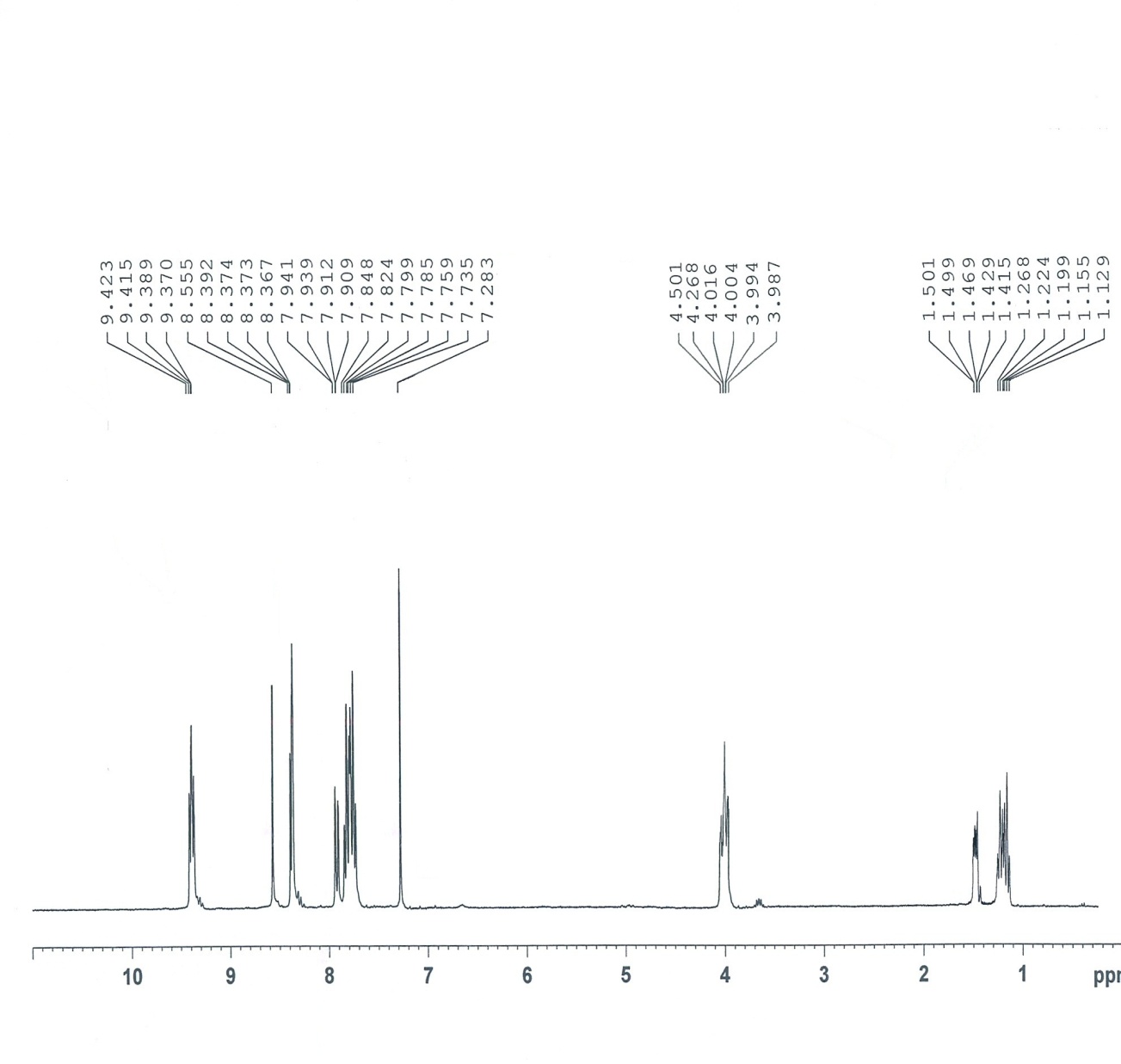

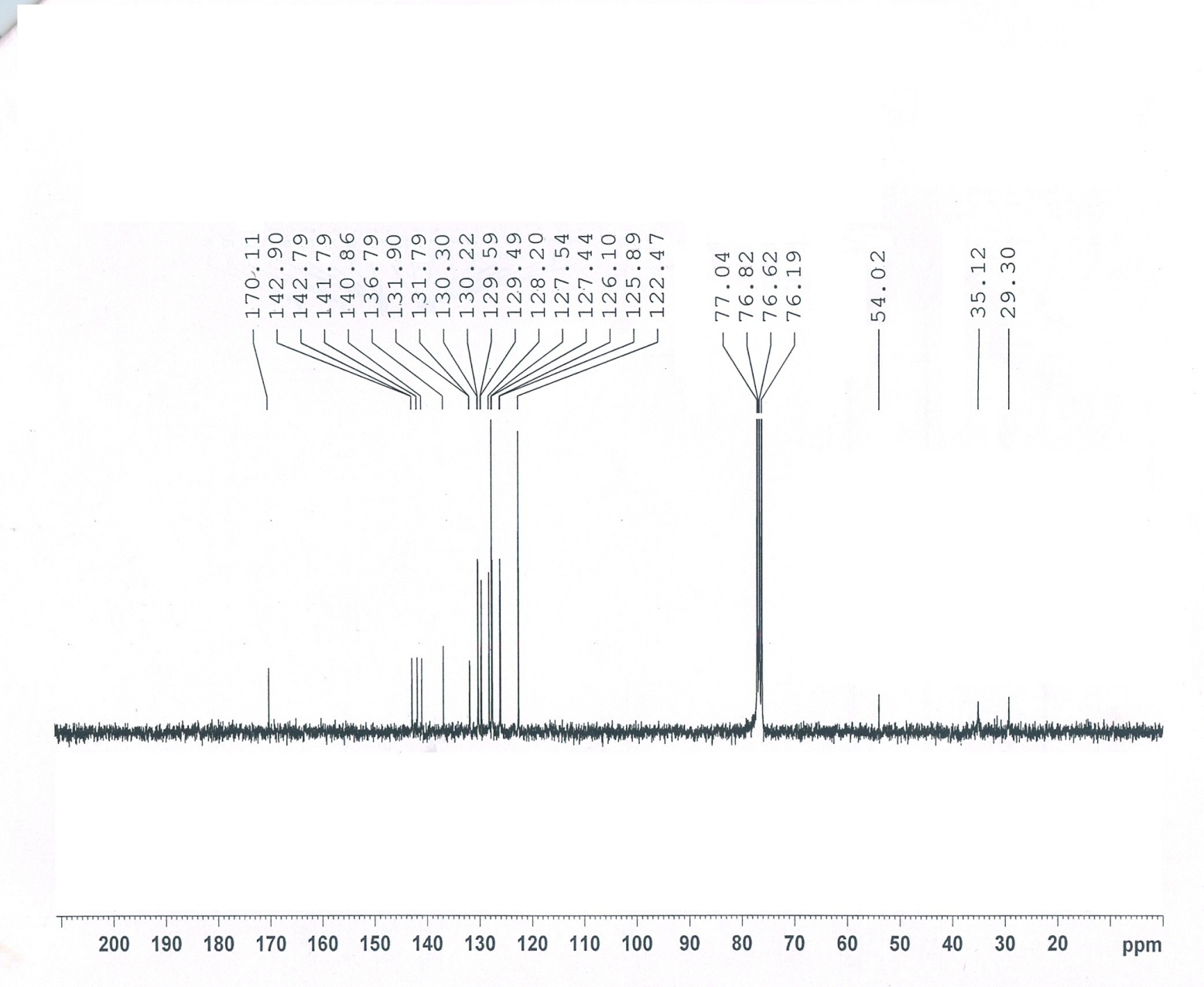
**

**
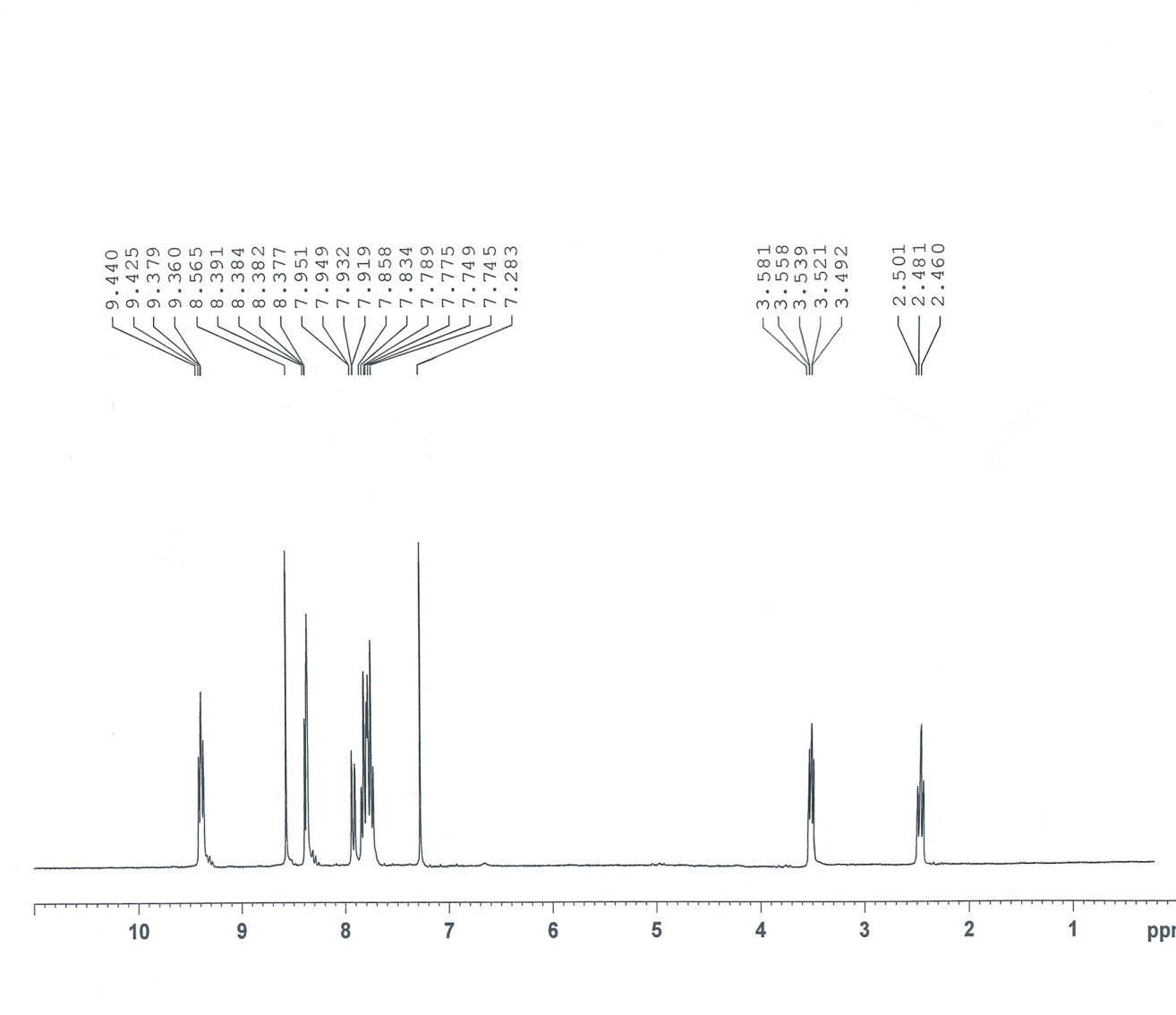
**


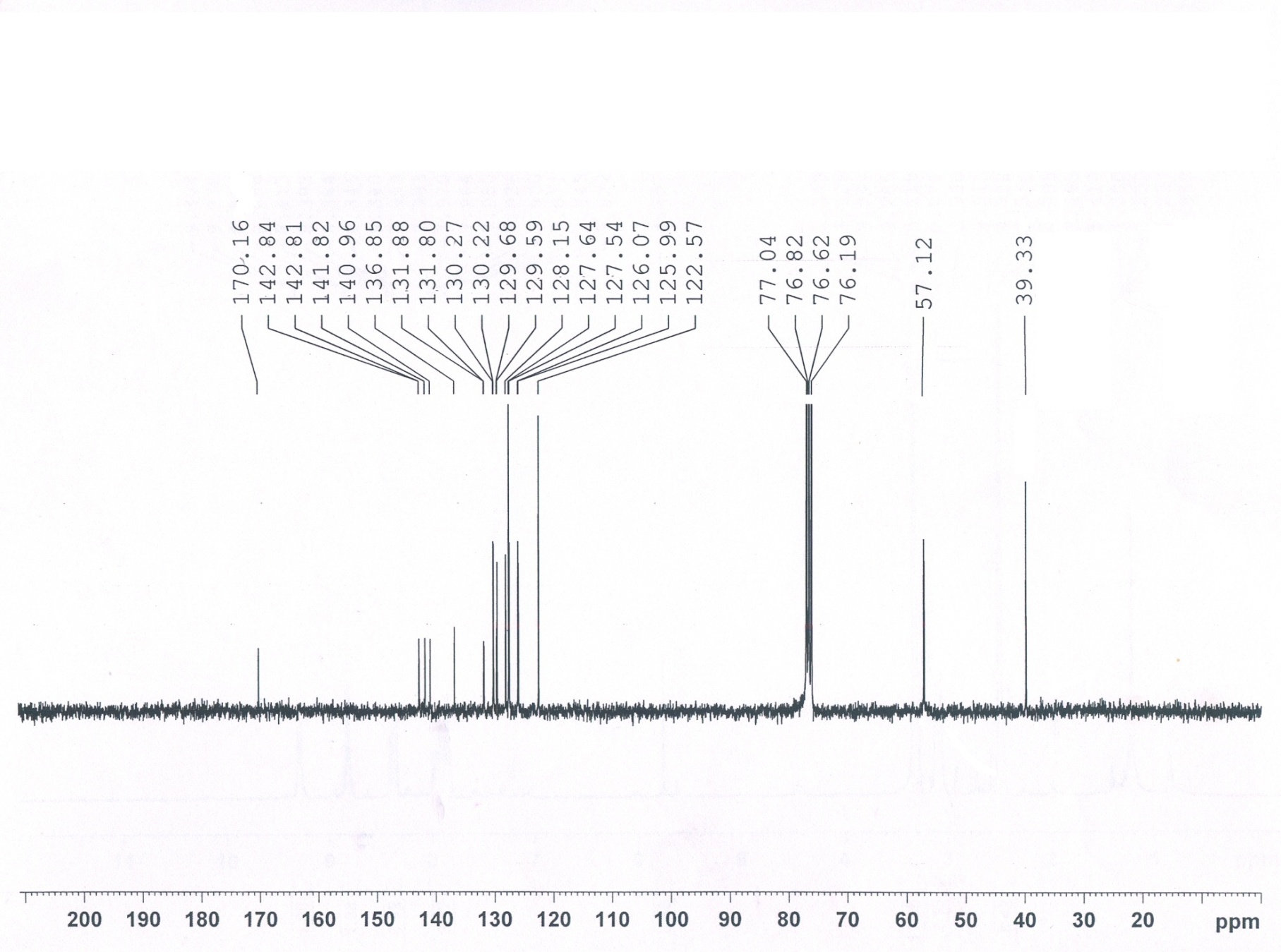


**
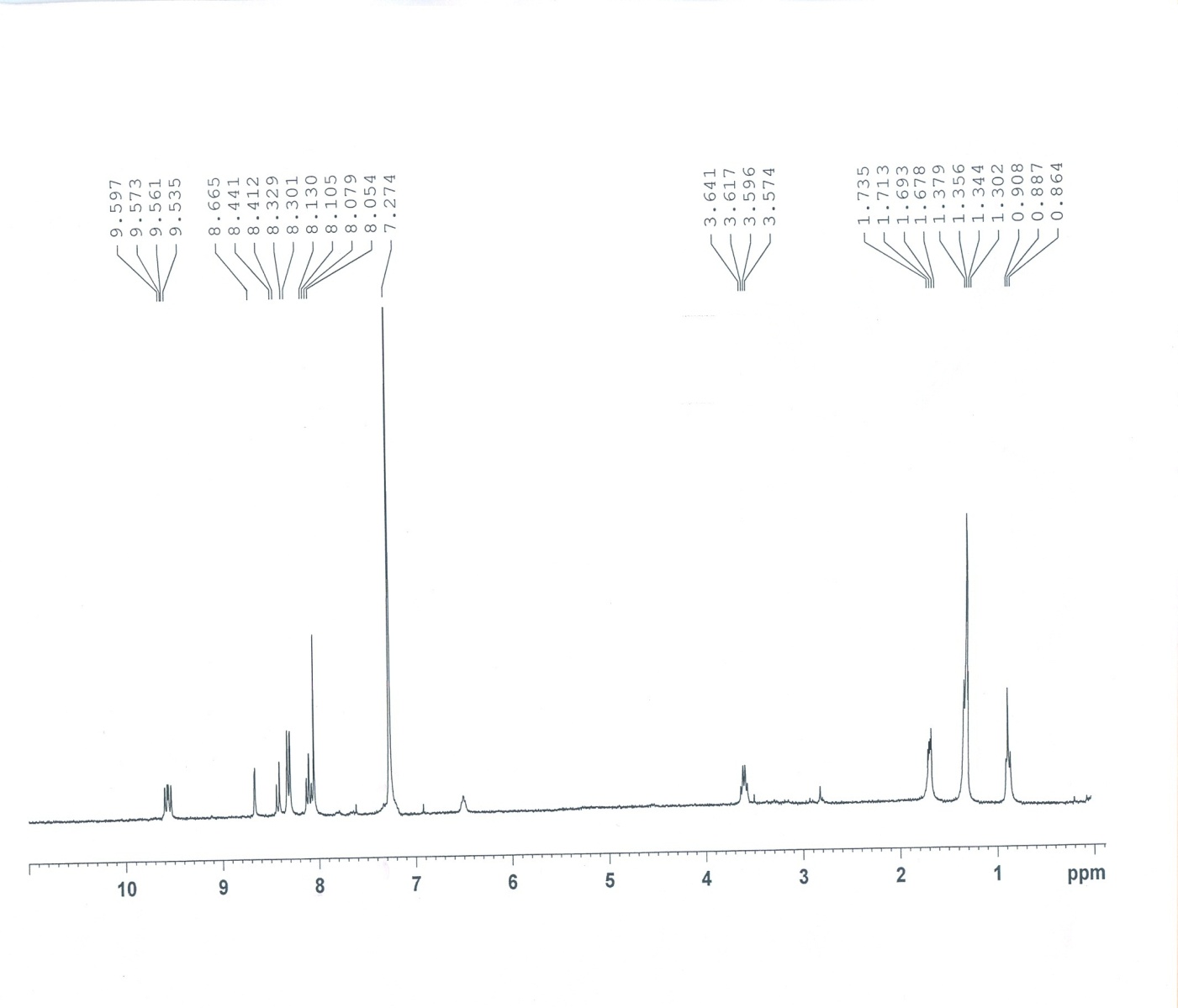
**

**
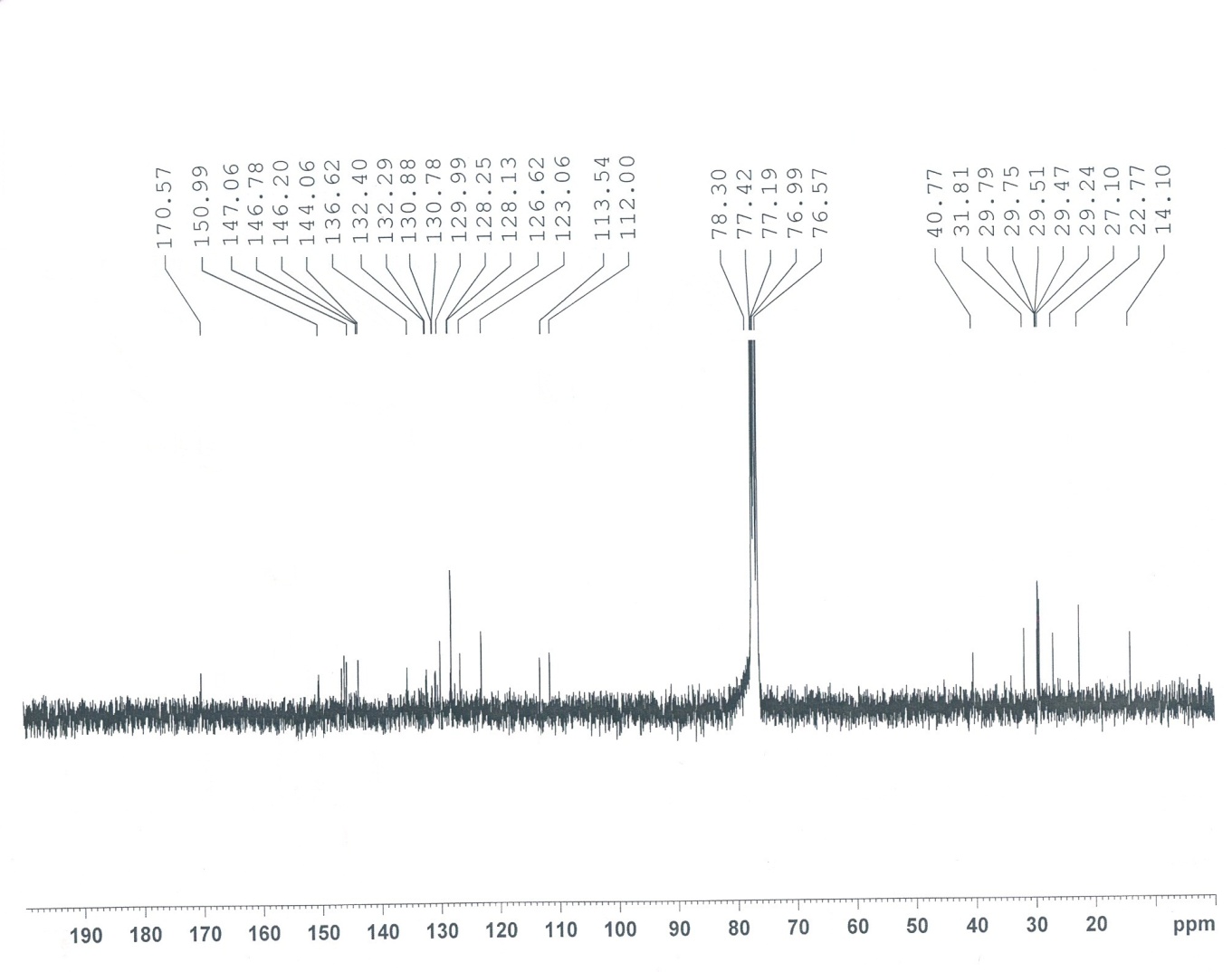
**

**6. Design and predicted packing of phenazine nanobuilding block (8) with Z-matrix**

Our entire calculations were performed at DFT/B3LYP using Gaussian 09 program package, invoking gradient geometry optimization as well as unit cell optimization. GaussView 5.0.8 molecular visualization program was used in our study. We achieved the stabilizing energy as -1001.0035 × 10³ Kcal/mol (SI Fig. 3). Among the numerous available DFT methods, we have selected the B3LYP method, which combines the Becke’s three parameter exchange functional (B3) with the Lee, Young and Parr correlation functional (LYP). DFT calculation enabled us to find out the expected molecular packing of the compound **8** (SI Fig. 4) and its higher order (SI Fig. 5). The computational study for the optimized molecular structural parameters and HOMO–LUMO energy gap (SI Fig. 6) for **8** have been investigated using B3LYP/6-31G basis set and it was found as 0.1210 eV (SI Fig. 7). Our calculated results have shown that the investigated framework **8** is polar in nature and possesses a dipole moment of 4.0823 Debye. We achieved the stabilizing energy as -1001.0035 × 10³ Kcal/mol. In the energy minimized molecular structure of **8** shows flat sheet like structure. From higher order packing along *b*-axis it has been observed that hydrogen bonding and π-π interaction is the main factor, which helps to form a parallel beta sheet like self-assembled structure.


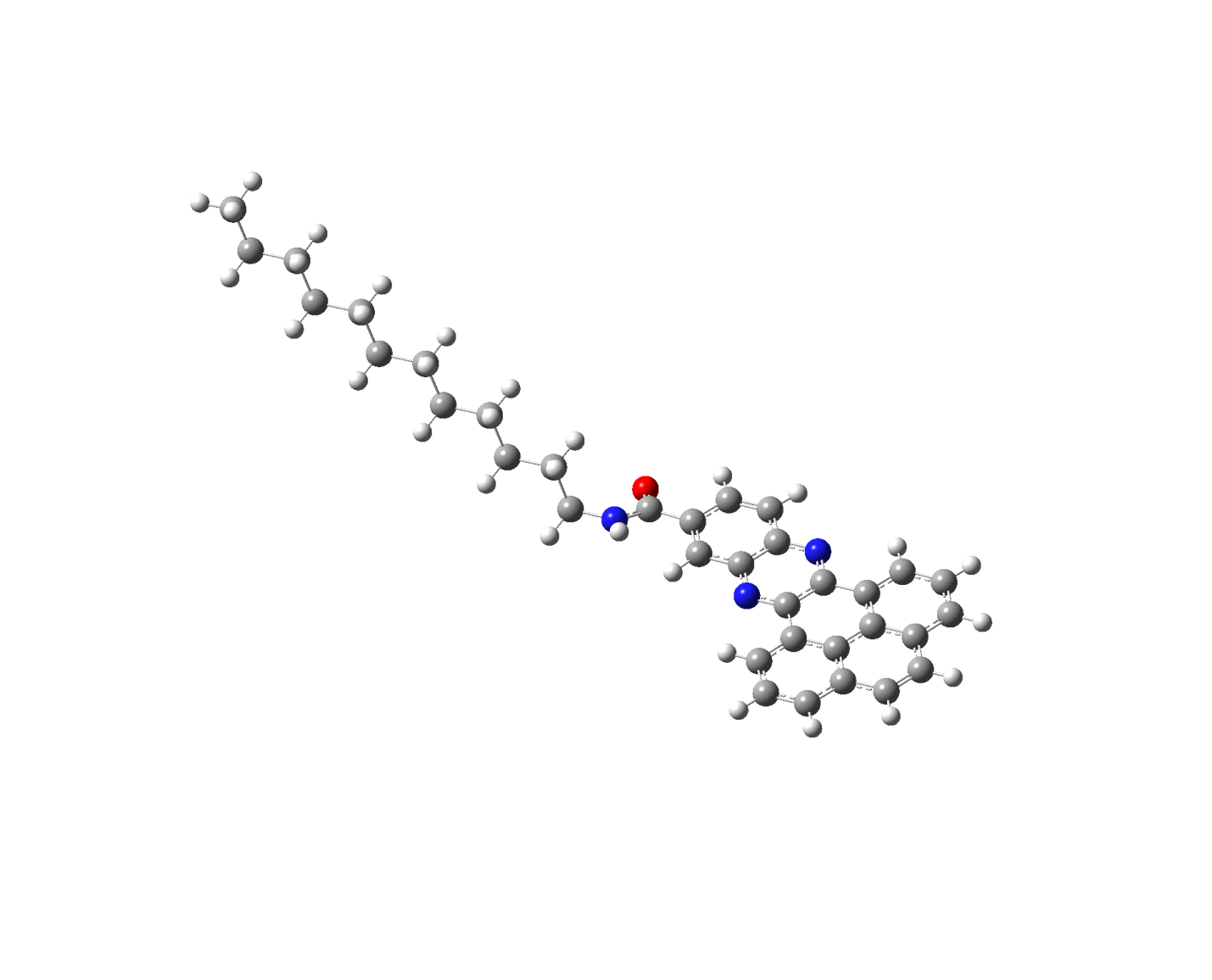


**SI Figure 3│**Optimized structure of **8** using B3LYP/6-31G basic set


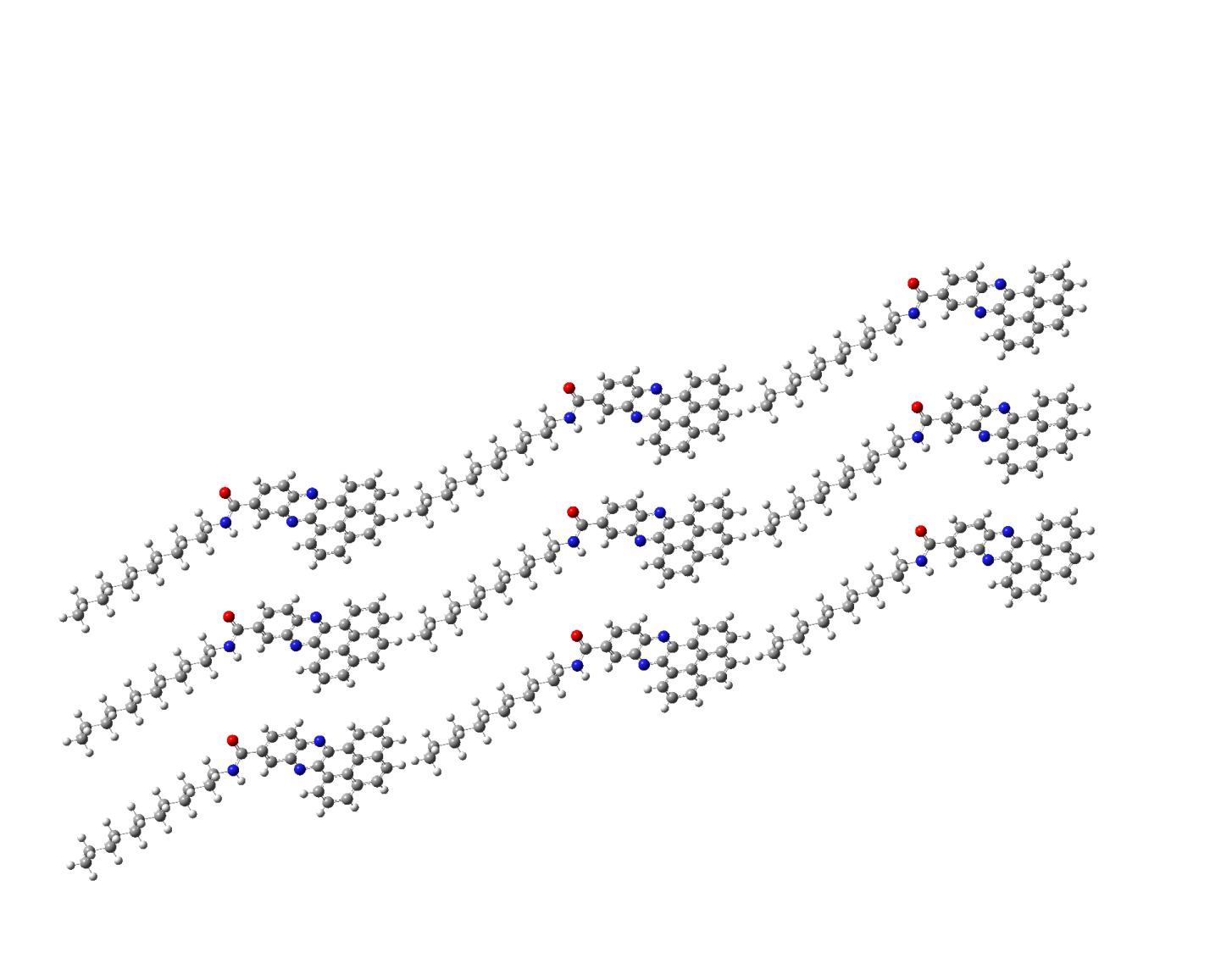


**SI Figure 4│**Packing arrangement of compound **8** along *a* axis


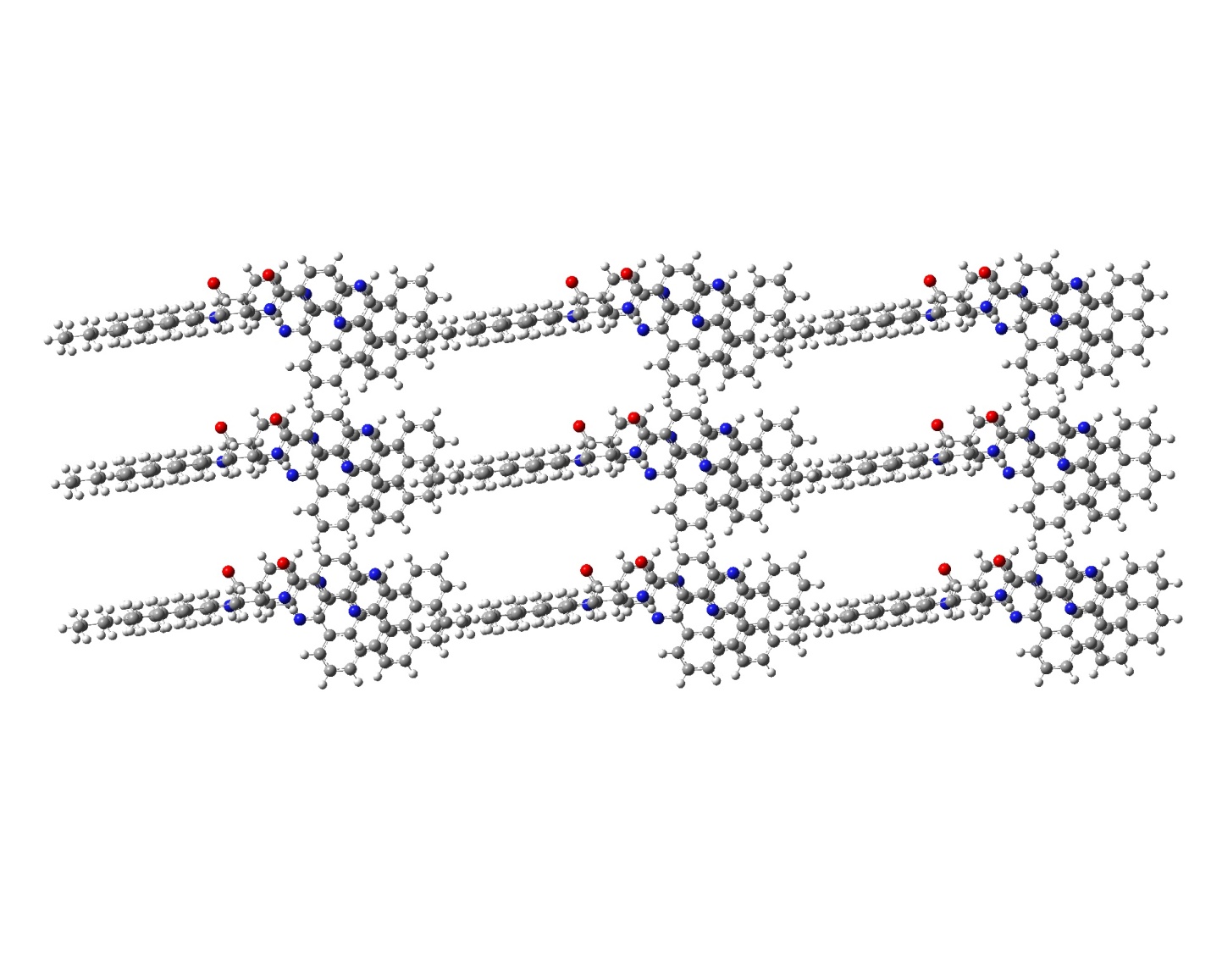


**SI Figure 5│**Higher order packing arrangement of compound **8**


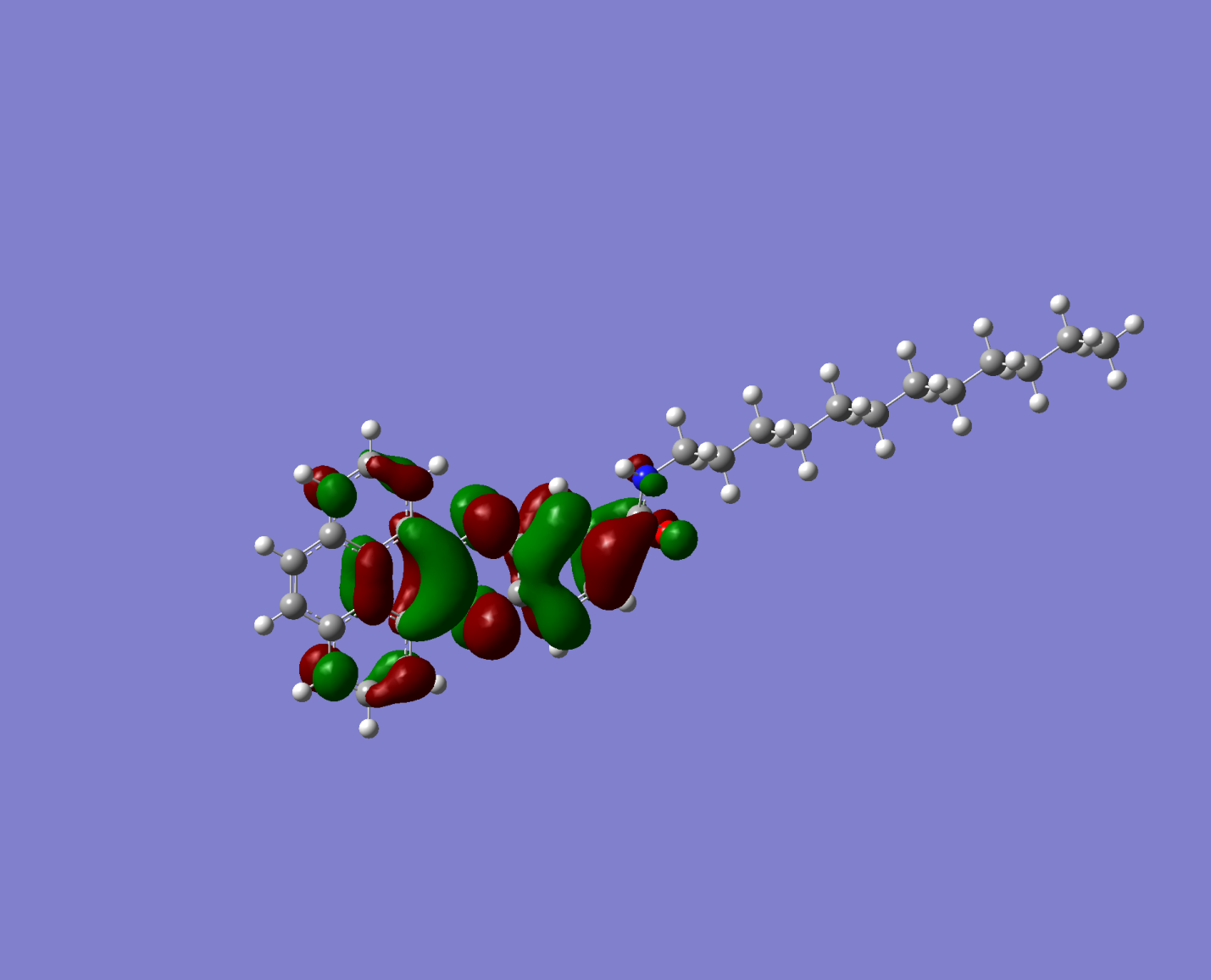


**SI Figure 6│**HOMO-LUMO diagram of compound **8**

**
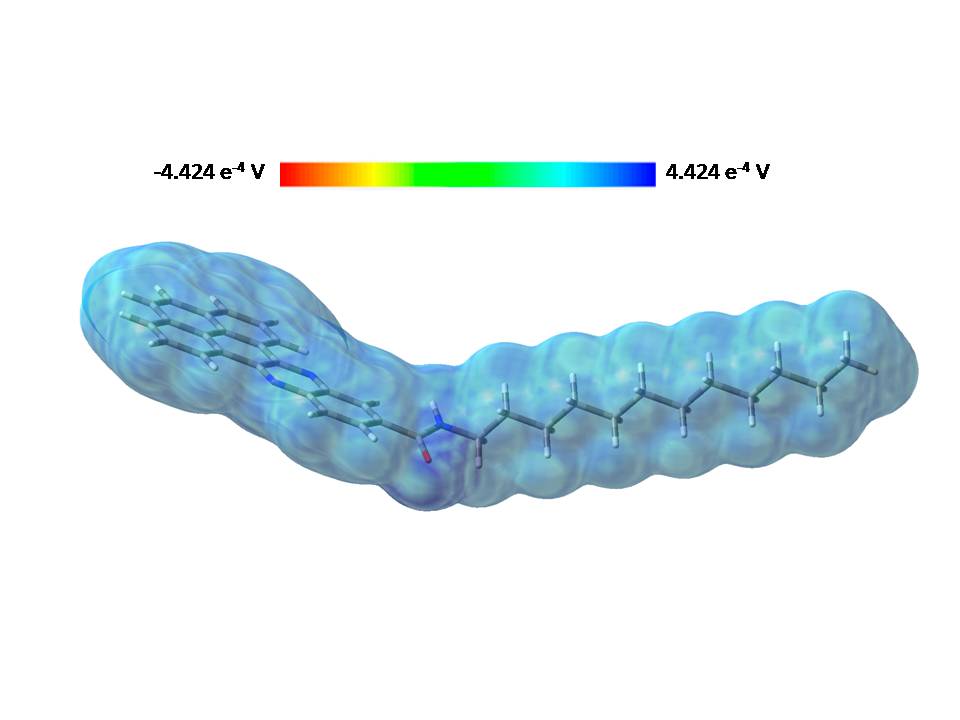
**

**SI Figure 7│**Total electron density mapping of compound **8**

**Optimized Z-matrix data of compound 8**

Center Atomic Atomic Coordinates (Angstroms)

Number Number Type X Y Z

---------------------------------------------------------------------

1 6 0 -6.310114 3.827285 -1.024577

2 6 0 -7.619359 3.769220 -0.556051

3 6 0 -8.123160 2.591047 0.036116

4 6 0 -7.269479 1.450487 0.151428

5 6 0 -5.931454 1.527254 -0.333481

6 6 0 -5.468198 2.712823 -0.914915

7 6 0 -9.473346 2.506339 0.527280

8 6 0 -7.770795 0.241648 0.753817

9 6 0 -9.117465 0.193576 1.230016

10 6 0 -9.948277 1.361300 1.097022

11 6 0 -9.596150 -0.997411 1.817449

12 1 0 -10.620176 -1.030860 2.177888

13 6 0 -8.774047 -2.114331 1.935481

14 6 0 -7.452965 -2.074054 1.471052

15 6 0 -6.942537 -0.911182 0.882804

16 1 0 -10.110028 3.380986 0.432949

17 1 0 -5.936890 4.739677 -1.478030

18 1 0 -8.270575 4.634195 -0.641901

19 1 0 -4.447633 2.745189 -1.275980

20 1 0 -10.969284 1.309755 1.463311

21 1 0 -9.156132 -3.022881 2.389136

22 1 0 -6.803083 -2.936244 1.556221

23 6 0 -1.669546 -0.626915 -1.014085

24 6 0 -3.007363 -0.672531 -0.543230

25 6 0 -3.506235 -1.875863 0.054275

26 6 0 -2.651309 -3.007418 0.154313

27 6 0 -1.359402 -2.937815 -0.313117

28 6 0 -0.848375 -1.734583 -0.892024

29 1 0 -1.351073 0.293231 -1.491790

30 1 0 -3.052452 -3.909642 0.601332

31 1 0 -0.692378 -3.789982 -0.263772

32 6 0 -5.566965 -0.854121 0.392876

33 6 0 -5.065274 0.355878 -0.211160

34 7 0 -3.807320 0.427169 -0.666321

35 7 0 -4.788952 -1.938741 0.514607

36 6 0 0.570985 -1.768182 -1.375625

37 8 0 1.152975 -2.861351 -1.588995

38 6 0 2.579597 -0.474528 -2.042623

39 1 0 2.777468 -1.389034 -2.608002

40 1 0 2.650129 0.376165 -2.732380

41 6 0 3.609656 -0.327983 -0.908016

42 1 0 3.368492 0.561388 -0.306405

43 1 0 3.520887 -1.196534 -0.242363

44 6 0 5.050810 -0.217790 -1.436945

45 1 0 5.288541 -1.113197 -2.030684

46 1 0 5.123923 0.638181 -2.126128

47 6 0 6.096956 -0.057313 -0.319041

48 1 0 5.858247 0.837939 0.275569

49 1 0 6.025069 -0.912549 0.369853

50 6 0 7.539805 0.052336 -0.844464

51 1 0 7.780179 -0.845199 -1.434660

52 1 0 7.609457 0.904264 -1.538512

53 6 0 8.585941 0.221254 0.272104

54 1 0 8.344041 1.118071 0.863180

55 1 0 8.517606 -0.631050 0.965554

56 6 0 10.028903 0.333899 -0.252555

57 1 0 10.272127 -0.564012 -0.841243

58 1 0 10.096245 1.184573 -0.948318

59 6 0 11.074592 0.507780 0.863680

60 1 0 10.829960 1.404833 1.453255

61 1 0 11.008586 -0.343510 1.558693

62 6 0 12.517471 0.623633 0.339401

63 1 0 12.762982 -0.274143 -0.248680

64 1 0 12.582823 1.473929 -0.357040

65 6 0 13.562755 0.800745 1.455345

66 1 0 13.317545 1.698198 2.044265

67 1 0 13.499173 -0.049723 2.151813

68 6 0 15.006046 0.918334 0.931691

69 1 0 15.250777 0.021113 0.344039

70 1 0 15.068960 1.767500 0.235096

71 6 0 16.042331 1.096520 2.052798

72 1 0 16.023154 0.246103 2.746121

73 1 0 17.058776 1.176921 1.650175

74 1 0 15.840088 2.004189 2.635426

75 7 0 1.196883 -0.564927 -1.562599

76 1 0 0.742185 0.282440 -1.258078

**Optimized matrix data of compound 8-unit cell**

Center Coordinates (Angstroms)

Number X Y Z

---------------------------------------------------------------------

C 8.20724910 8.65432496 2.21355908

C 6.89750110 8.59637296 2.68069208

C 6.39274110 7.41800196 3.27164808

C 7.24596410 6.27711996 3.38716508

C 8.58450010 6.35376296 2.90364808

C 9.04870910 7.53953796 2.32340108

C 5.04203110 7.33341196 3.76138408

C 6.74368310 5.06808596 3.98835708

C 5.39651710 5.02014596 4.46316608

C 4.56618410 6.18818796 4.32999208

C 4.91689110 3.82897796 5.04946208

H 3.89249310 3.79562996 5.40885708

C 5.73855510 2.71176196 5.16773108

C 7.06011510 2.75190496 4.70465208

C 7.57147310 3.91494096 4.11753708

H 4.40571110 8.20830696 3.66692708

H 8.58121910 9.56688096 1.76105108

H 6.24663210 9.46159496 2.59469408

H 10.06965410 7.57181296 1.96340108

H 3.54479610 6.13673896 4.69522808

H 5.35575410 1.80307896 5.62051508

H 7.70966510 1.88948296 4.79001708

C 12.84610710 4.19839496 2.22511008

C 11.50794710 4.15300696 2.69502208

C 11.00815010 2.94952196 3.29143108

C 11.86261710 1.81762796 3.39147708

C 13.15486310 1.88701596 2.92495808

C 13.66675710 3.09033996 2.34697908

H 13.16507710 5.11885996 1.74833308

H 11.46090310 0.91529496 3.83776408

H 13.82157110 1.03460596 2.97434408

C 8.94754510 3.97184696 3.62898808

C 9.45018910 5.18203796 3.02610208

N 10.70854410 5.25312796 2.57202708

N 9.72504410 2.88686596 3.75072508

C 15.08638710 3.05627596 1.86414008

O 15.66814710 1.96285696 1.65134208

C 17.09532110 4.34910796 1.19662608

H 17.29311610 3.43393696 0.63228608

H 17.16581410 5.19898796 0.50584808

C 18.12556410 4.49695296 2.33087108

H 17.88445110 5.38691496 2.93162708

H 18.03707310 3.62909696 2.99747008

C 19.56656810 4.60678896 1.80142208

H 19.80400210 3.71115296 1.20789708

H 19.63950510 5.46250396 1.11191008

C 20.61315610 4.76752496 2.91886508

H 20.37454510 5.66274896 3.51355708

H 20.54175910 3.91229196 3.60780608

C 22.05574210 4.87746896 2.39276108

H 22.29596610 3.98005996 1.80231008

H 22.12490610 5.72950396 1.69880108

C 23.10241310 5.04638796 3.50882608

H 22.86056510 5.94296996 4.10028108

H 23.03471710 4.19386596 4.20206508

C 24.54505310 5.15962696 2.98340008

H 24.78823310 4.26196396 2.39431608

H 24.61172810 6.01051496 2.28783708

C 25.59131710 5.33351996 4.09909508

H 25.34671110 6.23030696 4.68908608

H 25.52601710 4.48199096 4.79387808

C 27.03385110 5.45004096 3.57401308

H 27.27934410 4.55253196 2.98551708

H 27.09848410 6.30056196 2.87778408

C 28.07971810 5.62718496 4.68940808

H 27.83453210 6.52437996 5.27873408

H 28.01684710 4.77648996 5.38566008

C 29.52266710 5.74543496 4.16495808

H 29.76737810 4.84848396 3.57688508

H 29.58487910 6.59483496 3.46858908

C 30.55951510 5.92361996 5.28555008

H 30.54097810 5.07299196 5.97863008

H 31.57571210 6.00447096 4.88239408

H 30.35728510 6.83105396 5.86854908

N 15.71266610 4.25923196 1.67687208

H 15.25804510 5.10693396 1.98056708

Tv 29.03091600 0.00000000 0.00000000

Tv 0.00000000 9.65158600 0.00000000

Tv 0.00000000 0.00000000 6.47278200

**7. Morphology of organic nanomaterials of pure 4-8 and mixed 8+SA**

The synthesized phenazines (**4**-**8**) possessing lower symmetry elements and several noncovalent weak binding forces led to fabrication of unidirectional packing nanomaterials, such as rods (**4**), flowers (**5**) and flake-assemblies (**6**) through nano-crystallization, nanoballs (**7**) by spin coating, ultra-long nanofibrils (**8**) from drop-casting, and small fibers of mixed on drop casting (SI Fig. 9). The following are the one-dimensional (1D) morphologies found in scanning electron microscope (SEM) imaging. TEM images of the small constituting nanoparticles (~5-10nm) of nanofibrils for ***sample 1*** (3:1; RRAM) and ***sample 2*** (1:3; WORM) are shown in SI Fig. 10.

**
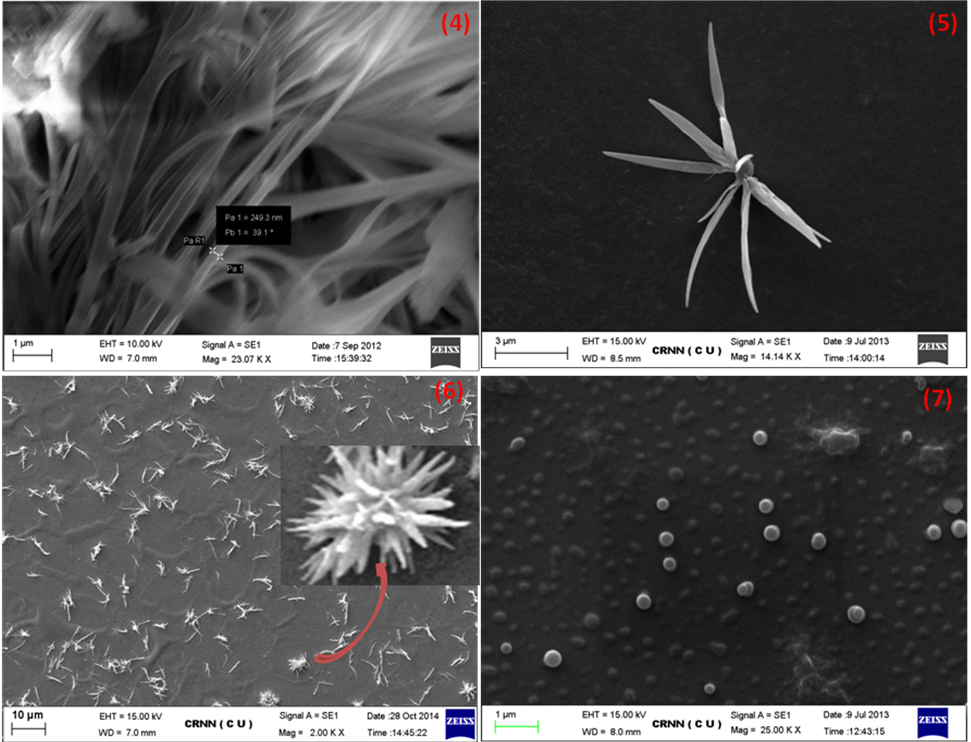
**

**
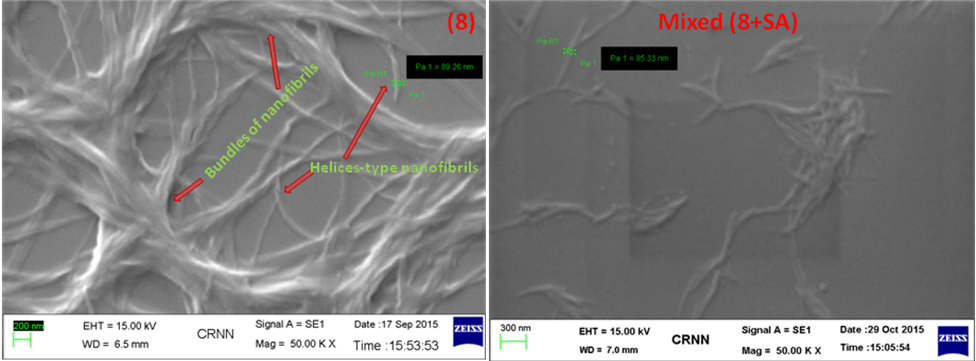
**

**SI Figure 9│**SEM images of nanomaterials (**4**-**8**) and mixed **8** + **stearic acid** (1:1)

**SI Figure 10│**TEM images of constituting nanoparticles of nanofibrils

**8. Isotherm measurement and film formation**

A Langmuir-Blodgett (LB) film deposition instrument (Apex 2000C, Apex Instruments Co.) was used for surface pressure–area isotherm measurements and monolayer film preparation. Ultra pure Milli-Q water of resistivity 18.2 MΩ–cm was used as subphase. The concentration of the stock solutions for both stearic acid (**SA**) and **8** was 0.5 mg/mL. In order to measure the isotherm and film preparation, 60 micro liters of either pure or mixed solutions were spread onto the subphase with a micro syringe. After complete evaporation of volatile solvent, barrier was compressed at a rate of 5 mm/min. to record the surface pressure–area per molecule isotherms. The surface pressure (π) versus average area available for one molecule (A) was measured by a Wilhelmy plate arrangement.^1^ Each of the isotherms was repeated a number of times and data for surface pressure–area per molecules isotherms were obtained by a computer interfaced with the LB instrument. Smooth fluorescence grade quartz plates for spectroscopy and Si-wafer for atomic force microscopic (AFM) studies were used as solid substrate. Y-type deposition at a particular surface pressure was followed to transfer Langmuir films at a deposition speed of 5 mm/min. For AFM measurement, a single layer was deposited.

Reference

**Excess area analysis**

The excess area of the mixed monolayer have been calculated by using the relation,

A_E_ = A_12_- A_id_

with A_id_=A_1_X_1_+A_2_X_2_, where A_id_ is the ideal area per molecule, A_1_ and A_2_ are the areas occupied by the monomers of SA and **8** respectively and X_1_ and X_2_ are the mole fractions of the components in the mixtures. A_12_ is the experimentally observed area per molecule.

Ideally the plot of A_E_ versus X_2_ should be a straight line. Any deviation from it (A_E_ = A_12_- A_id_ ≠ 0) indicates partial miscibility and non-ideality.^2^ If the intermolecular forces between the components of the mixed monolayer are attractive, A_E_ will be negative. On the other hand, positive value (A_E_>0) reveals a repulsive interaction among the constituent components of the mixed monolayer.

Reference

**Gibbs free energy analysis**

The excess Gibbs free energy of mixing ($\Delta G_{exc}$) have been calculated by using the relation.^3^

$$\Delta G_{exc}=N\int_{0}^{\pi} [A_{12}-(A_{1}X_{1}+A_{2}X_{2})]d\pi$$

where $A_{12}$ is the experimentally observed area per molecule, $A_{1}$ and $A_{2}$ are the areas per molecule of the individual components at molefractions $X_{1}$ and $X_{2}$, N is Avogadro's number. The results obtained from the surface phase rule.^4^ The excess Gibbs free energy $\Delta G_{exc}$ gives the information about the interactions between the components of a mixed monolayer system. The value of $\Delta G_{exc}$ reveals the information whether the particular interaction is energetically favourable ($\Delta G_{exc}$ <0) or not ($\Delta G_{exc}$ >0), while for $\Delta G_{exc}$= 0 ideal mixing takes place i.e. no interaction takes place between the components.

Reference

9. **BAM experiments**

The morphology of the film at the air-water interface was observed using a Brewster Angle Microscope equipped with a 30 mW laser emitting p-polarized light at 532 nm wavelength, which was reflected off the air/water interface at the Brewster angle (53.1°). This reflected beam pass through a focal lens, into an analyzer at a known angle of incident polarization, and finally to a CCD camera, which measures gray level instead of relative intensity. The lateral resolution of the microscope was 2 μm, the shutter speed was 1/50 s and the images were digitalized.

From the BAM image of pure **8** monolayer (SI Fig.11), it is seen that phenazine molecules formed almost smooth uniform monolayer at air-water interface. On the other hand BAM images of mixed monolayer (SI Fig.12,13) showed that domains are formed due to interaction between the constituent **8** and **SA** molecule in the mixed films. However nature of domain formation is little bit different for lower and higher molefraction of **8**. This is because at lower mole fraction (0.1 – 0.3) strong binding ability between **SA** and **8** molecules is observed (Fig. 2b), whereas, mole fraction greater than 3 interaction between **SA** and **8** molecules balance each other. As a whole BAM study give compelling visual evidence of the formation of monolayer at air-water interface.


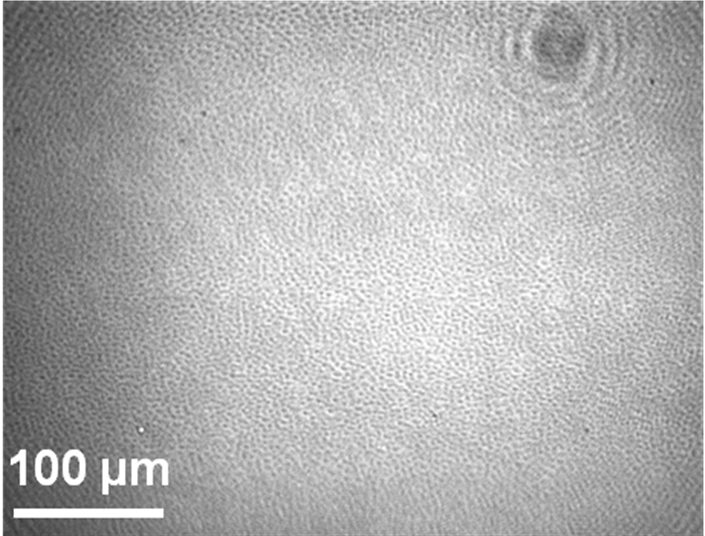


**SI Figure 11│**BAM image of pure **8** monolayer


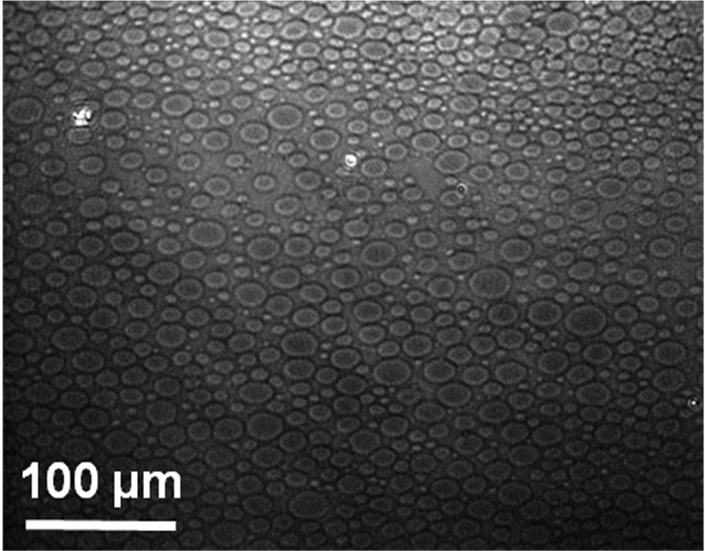


**SI Figure 12│** BAM image of **8-SA** mixed monolayer (X**_8_** =0.2 M)


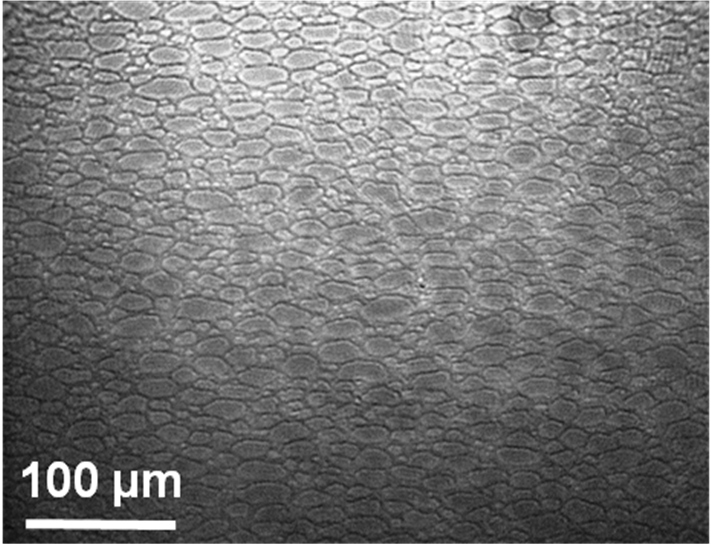


**SI Figure 13│** BAM image of **8-SA** mixed monolayer (X**_8_** =0.5 M)

**10. UV-Vis study**

UV-Vis study were performed using dilute solution of **8** in methanol and solid state LB films of pure **8** and mixed **8** and stearic acid (1:1). The photophysical studies of the pure **8** and mixed **8**-**SA** LB films showed red-shifted and broadened UV-vis spectra, which confirmed the existence of J-type aggregation.^5^

**SI Figure 9│** Normalized UV−vis absorption spectra of dilute **8** in solution, pure **8** and **8**-SA mixed LB films

**11. References**

1. Ulman, A. An introduction to ultrathin organic films: from Langmuir–Blodgett films of self ssemblies; Academic Press, New York, 1991.
2. Hsu, W.-P., Chiou, M.-S., & Li, H.-Y. Miscibility of mixed stereoregular PMMA/PVCN monolayers at the air/water interface. *J. Appl. Polym. Sci.* **124**, 333–341 (2012).
3. Dynarow-Lątka, P.; Kita, K. Molecular Interaction in Mixed Monolayers at the Air/Water Interface. *Adv.* *Colloid Interface Sci.* **79**, 1−17, (1999).
4. Gaines, G. L. Jr. *Insoluble Monolayers at Liquid-Gas Interface*; John Wiley & Sons: New York, 1966.
5. Debnath, P., Chakraborty, S., Deb, S., Nath, J., Bhattacharjee, D. & Hussain, S.A. Reversible Transition between Excimer and J‑Aggregate of Indocarbocyanine Dye in Langmuir−Blodgett (LB) Films. *J. Phys. Chem. C.* **119**, 9429-9441 (2015).
